# The Synthesis of Biphasic Metabolites of Carfentanil

**DOI:** 10.3390/molecules28227625

**Published:** 2023-11-16

**Authors:** Junchang Wang, Jianwen Hu, Pingyong Liao, Shang Xue, Shan He, Ruijia Chen, Xuejun Zhao, Wenbin Liu

**Affiliations:** 1 Shanghai Yuansi Standard Science and Technology Co., Ltd., Shanghai 200072, China; 2 Shanghai Key Laboratory of Crime Scene Evidence, Shanghai Research Institute of Criminal Science and Technology, Shanghai 200072, China

**Keywords:** carfentanil, metabolism, carbohydrate, glycoconjugates

## Abstract

Carfentanil is an ultra-potent synthetic opioid. The Russian police force used both carfentanil and remifentanil to resolve a hostage incident in Moscow. This reported use sparked an interest in the pharmacology and toxicology of carfentanil in the human body, and data on its metabolites were later published. However, there have been few studies on the synthesis of carfentanil metabolites, and biological extraction has also put forward large uncertainty in subsequent studies. The aim of the present study is to investigate the synthesis of biphasic metabolites that are unique to carfentanil. The purpose was to produce corresponding metabolites conveniently, quickly, and at low cost that can be used for comparison with published structures and to confirm the administration of carfentanil.

## 1. Introduction

Carfentanil is an ultra-potent synthetic opioid analgesic that belongs to the same class of drugs as its prototype fentanyl. Although carfentanil has not been used in human clinical medicine, it is used in veterinary medicine for the sedation, hypotension, and anesthesia of large animals [1]. Measurements taken by the tail withdrawal test on rats have shown that the analgesic effect of carfentanil is 10,000 times stronger than that of morphine [2]. Carfentanil has the lowest EC50 value of the fentanyl analogues [3]. The illegal use of carfentanil has increased worldwide, with many records of unintentional deaths [4]. Also, a study confirmed the fatal use of aerosolized carfentanil and remifentanil by the Russian Defense Forces in the settlement of a 2002 terrorist siege [5].

The pharmacological properties of carfentanil are like those of fentanyl. Carfentanil selectively binds to the μ-opioid receptor (MOR) [6], thereby predominantly acting on the respiratory and central nervous systems. The binding affinity of the μ-opioid receptors of carfentanil is 20 times higher than that of fentanyl [7], and its high lipophilicity and high protein binding ability are believed to be the reasons for its high potency. After binding to the μ-opioid receptors in rodents, carfentanil shows not only opioid effects such as analgesia and pleasure but adverse reactions, such as muscle stiffness and respiratory inhibition, can also be present [8,9], which are an important cause of death. Carfentanil is mainly used as an anesthetic for large animals, and as a radioactive tracer for positron emission tomography imaging after (^11^C labeling) [10,11]. Carfentanil has not yet been used for human clinical treatment. It has undergone some pharmacological studies on animals, with only a small amount of data reported from human studies [12].

Unfortunately, carfentanil has invaded the field of the street drug market in the last decade and, thereby, caused countless fatal overdoses and deaths through respiratory arrest and pulmonary edema. Because of this situation, all scientific studies that aim to obtain new knowledge about carfentanil and 4-anilidopiperidine must be considered as important.

**Figure 1:**
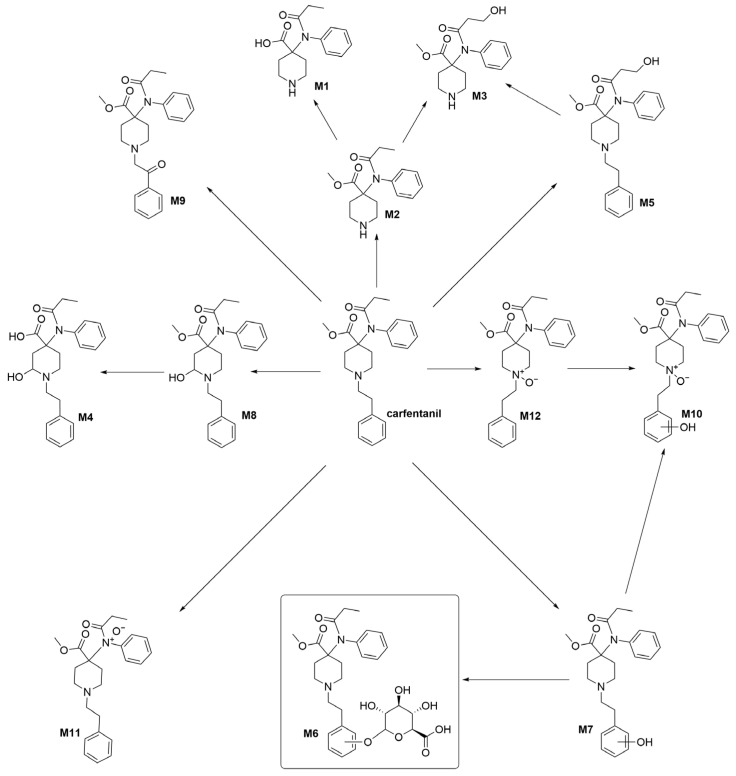
Proposed metabolic pathways for carfentanil in human hepatocytes.

In 2016, Kristovich and Huestis published a study on the metabolites of carfentanil [13], in which they used MS/MS and ion chromatography analyses to derive their structures (Figure 1). There are mainly 12 types of metabolites, among which a unique glycoconjugate has been detected. This compound can serve as a characteristic marker for the administration of carfentanil and has been used to distinguish between different types of fentanyl. However, the main source of the characteristic metabolites of carfentanil is currently non-synthetic, which is inefficient and has a significant impact on subsequent structural identification and analysis. In the present study, the key technologies for the development of biphasic metabolite standards for alfentanil were investigated, which were based on the metabolic pathways of carfentanil. In addition, the syntheses of high-purity biphasic metabolite reference materials have also been investigated, with the purpose of providing technical support and judgment basis for the abuse control and poisoning diagnosis of carfentanil.

## 2. Results and Discussion

**Figure 2:**
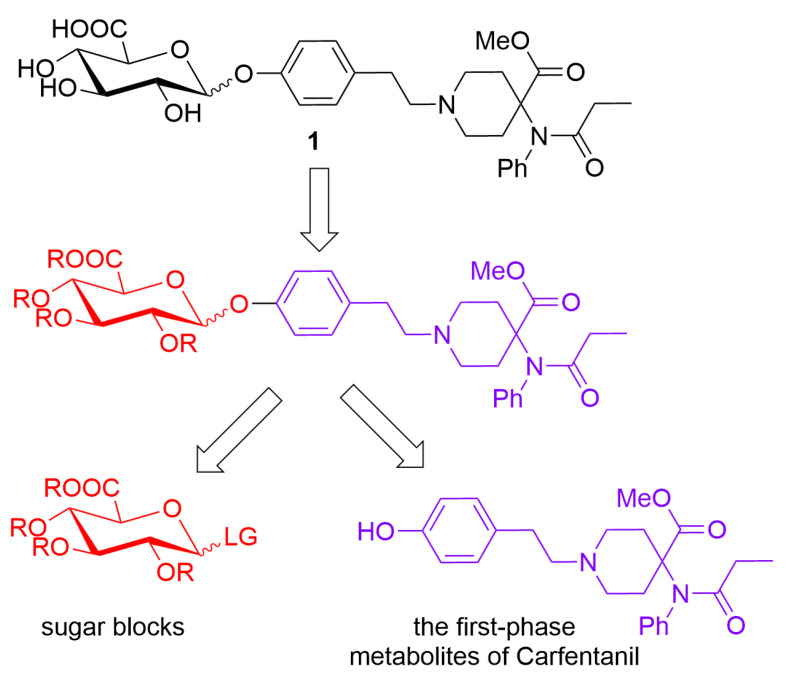
Retrosynthetic analyses of biphasic metabolites of carfentanil.

According to the retrosynthetic analyses, the synthesis of biphasic metabolites of carfentanil requires the construction of two types of blocks: sugar blocks and the first-phase metabolites of carfentanil. The two blocks are then linked by glycosylation reactions and finally deprotected to obtain the desired products (Figure 2).

**Figure 3:**
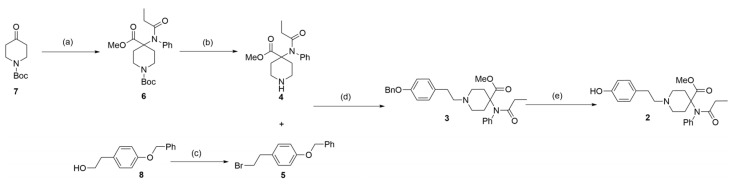
Synthesis of the first-phase metabolite (**2**) of carfentanil. Reagents and conditions: (**a**) 1. aniline, NaOH, and tetrahydrofuran (THF); 0 °C to room temperature for an overnight duration; yield of 53%. 2. Propionic anhydride, triethylamine, EA; 50 °C for an overnight duration. 3. Potassium carbonate, iodomethane, N, N-dimethylformamide (DMF); room temperature for an overnight duration; yield of 88% over two steps. (**b**) 4 M HCl/Dioxane and MeOH; room temperature for 2 h; yield of 95%; (**c**) CBr_4_, PPh_3_, and acetonitrile; 0 °C to room temperature for 2 h; yield of 89%. (**d**) Cesium carbonate and acetonitrile; 80 °C for an overnight duration; yield of 85%. (**e**) 10% Pd/C, acetic acid, H_2_, and MeOH; room temperature for an overnight duration; yield of 85%.

Based on previous studies on the synthesis of carfentanil [14,15,16], 4-piperidone (**7**) was used herein as the raw material. An ester (**6**) was then obtained in a three-step reaction, with a yield of 47%. Thereafter, t-butyloxy carbonyl was removed under acidic conditions to obtain fragment (**4**), which was linked with the brominated compound (**5**) by a nucleophilic attack. An ester (**3**) was, thereby, obtained with a yield of 85%. The linkage between fragment (**4**) and compound (**5**) was obtained by the Appel reaction using raw material (**8**). Finally, the benzyl was hydrogenated to obtain phenol (**2**), with a yield of 85%. This product is one of the first-phase metabolites of carfentanil (Figure 3). During this reaction process, thin-layer chromatography (TLC) and liquid chromatography–mass spectrometry (LC-MS) analyses were used for monitoring, and compound (**2**) was analyzed by nuclear magnetic resonance (NMR) and high-resolution mass spectroscopy (HRMS) (Figures S1–S11).

**Figure 4:**
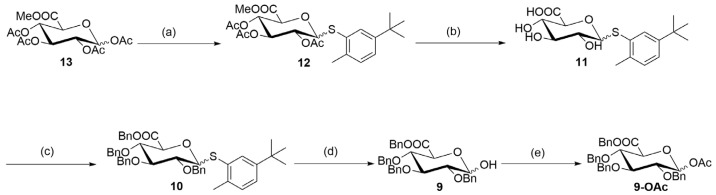
Synthesis of 1-hydroxyglucuronic acid (**9**). Reagents and conditions: (**a**) 5-tert-butyl-2-methylbenzenethiol, BF_3_.Et_2_O, and CH_2_Cl_2_; room temperature for 12 h; yield of 60%. (**b**) NaOH, MeOH, and THF; room temperature for 5 h. (**c**) 1. NaH, BnBr, and DMF; 0 °C to room temperature for an overnight duration. 2. TBAF, and THF; 0 °C to room temperature for 12 h; 62% over two steps. (**d**) N-bromosuccynimide (NBS), acetone, and H_2_O; room temperature for 5 h; yield of 98%. (**e**) Ac_2_O, 4-dimethylaminopyridine (DMAP), and CH_2_Cl_2_; room temperature for 6 h; yield of 78%.

Moreover, the commercially available tetra-acetylated glucuronic acid ester (**13**) was used as a raw material in the reaction with 2-methyl-5-*tert*-butylthiophene to obtain a glucoside (**12**), with a yield of 60%. Subsequently, the ester group in glucoside (**12**) was removed under alkaline conditions to obtain a carboxylic acid (**11**). In the following two-step process, the exposed hydroxyl and carboxyl groups in the carboxylic acid (**11**) were protected to obtain a benzyl ester (**10**), with a total yield of 62%. During this two-step process, some partially protected intermediates were formed that could be completely converted to the target fragment (**10**) through post-processing or recycling. Finally, the benzyl ester (**10**) was hydrolyzed to hydroxy (**9**) with a yield of 98% through a glycosylation reaction. This step was followed by acetylation protection to obtain ester (**9-OAc**) (Figure 4). The structure of ester (**9-OAc**) was unambiguously verified by analyzing the ^1^H and ^13^C, 2D NMR spectra at 89 ppm and 92 ppm for heterogeneous carbon and at 6.29 ppm and 5.78 ppm for heterogeneous hydrogen (Figures S19–S22). This result showed that the hydroxyl group of compound (**9**) is at position 1.

**Figure 5:**
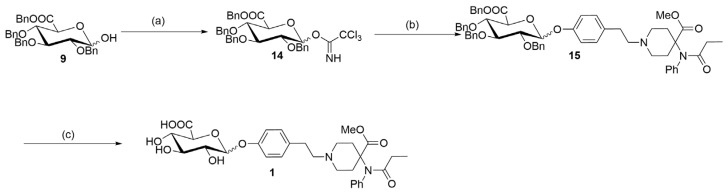
Synthesis of a biphasic metabolite of carfentanil (**1**). Reagents and conditions: (**a**) Trichloroacetonitrile, DBU, and CH_2_Cl_2_; 0 °C to room temperature for an overnight duration; yield of 80%. (**b**) compound (**2**), 4 Å MS, BF_3_.Et_2_O, and toluene; 0 °C to room temperature for 24 h; yield of 81%. (**c**) 10% Pd/C, acetic acid, H_2_, and MeOH; room temperature for an overnight duration; yield of 40%.

**Figure 6:**
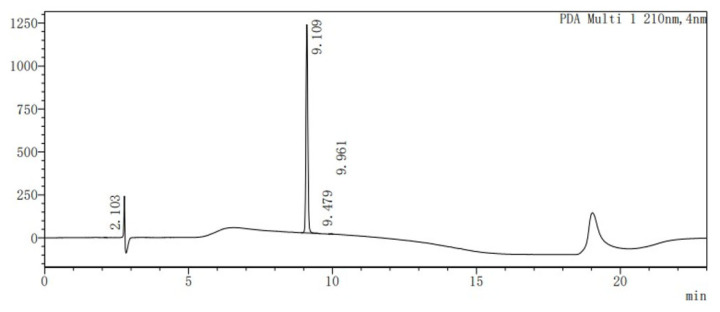
HPLC analysis of compound (**1**).

By using the two key fragments (**9**) and (**2**), the hydroxyl sugar was at first linked with trichloroacetate under alkaline conditions in an attempt to create a glycosyl donor. However, it was found that the use of anhydrous sodium carbonate resulted in almost no product formation. 1,8-diazabicyclo [5.4.0]undecane-7-ene (DBU) was instead used, with which it was possible to obtain the glycosyl donor (**14**) through column chromatography, with a yield of 80%. Through the use of the BF_3_.Et_2_O-catalyzed glycosylation reaction, ester (**15**) was then obtained, with a yield of 81% (Table 1). Finally, the benzyl group was removed by hydrogenolysis under heterogeneous catalytic conditions (H_2_, 10% Pd-C, and AcOH-MeOH) in the formation of the final product (**1**), with a yield of 40% (Figure 5). It was confirmed by HPLC analysis that the obtained purity of (**1**) was 99.6% (Figure 6). Also, the NMR analysis showed that there were basically no impurity peaks, which proved that the product was a high-quality reference substance. TLC and LC-MS analyses were used to monitor the reaction processes, and the formation of compound (**1**) was verified by NMR and HRMS analyses (Figures S24–S26).

## 3. Materials and Methods

### 3.1. General

Because of extremely high human safety hazards, “As little as 20 micrograms (an almost invisible drop) of carfentanil is lethal to humans” [17]. Therefore, experimental personnel should wear laboratory clothing, gloves, goggles, and masks when handling carfentanil. In addition, the experimental process should be completed in a fume hood. Also, when working with carfentanil derivatives, the reversing agent naltrexone hydrochloride must always be immediately available in the form of an injection.” All solvents and reagents that were used in this study were purchased from commercial sources and used without further purification. Also, TLC analyses were performed using SIL G/UV 254 silica glass plates. In addition, flash analyses were performed using Silica Gel 60 (200–400 mesh) and specific solvent chromatography systems (defined here in the experimental procedure for each synthesized molecule). Furthermore, NMR spectra were obtained using a JEOL JNM-ECZ600R 600 MHz spectrometer at room temperature. The chemical shifts were measured relative to the residual solvent peak of either CDCl3 (7.26 for ^1^H NMR and 77.16 for ^13^C NMR), DMSO-*d*6 (2.50 for ^1^H NMR and 39.52 for ^13^C NMR), or Methanol-*d*4 (3.31 for ^1^H NMR and 49.00 for ^13^C NMR). The ^1^H NMR data were reported in the following sequence: position (δ), multiplicity (s (singlet), d (doublet), t (triplet), q (quartet), dd (doublet of doublets), m (multiplet)), coupling constant (*J* in Hz), and relative integral. Also, the ^13^C NMR data were reported in the following sequence: position (δ) and multiplicity (m (multiplet)). In addition, the HRMS analyses were carried out by using a Vion IMS TOF-Q mass spectrometer, and deuterium incorporation was observed by this technique.

### 3.2. Experimental Section

#### 3.2.1. Synthesis of 1-(1,1-Dimethylethyl) 4-Methyl 4-[(1-Oxopropyl)phenylamino]-1,4-piperidinedicarboxylate (**6**)

NaOH (30 g, 750 mmol) was added at 0 °C to a solution of aniline (20 g, 215 mmol) in 600 mL of THF. After stirring for 10 min, compound **7** (90 g, 452 mmol) was added to the suspension. CHCl_3_ was thereafter added dropwise at 0 °C. The resulting suspension was then stirred at 0 °C for 1 h, after which the temperature was allowed to increase to room temperature, and stirring continued overnight. Thereafter, the suspension was filtered, and the filter cake was dissolved with H_2_O and then washed with tert-butyl methyl ether (MTBE). As the next step, the aqueous phase was acidified with concentrated HCl until a pH = value of 3, followed by extraction with CH_2_Cl_2_ (DCM). The organic layers were thereafter dried over Na_2_SO_4_ and concentrated to obtain a yellow solid (44.5 g, 53%). TEA (16.2 g, 160 mmol) was then added at room temperature to a solution of the yellow solid (17.2 g, 53.7 mmol) and propionic anhydride (20.9 g, 161 mmol) in 343 mL of EtOAc. The temperature of the resulting suspension was increased to 50 °C, and stirring continued overnight. Thereafter, the reaction mixture was neutralized with 1N HCl until a pH = value of 6 was reached, followed by extraction with EtOAc. The obtained organic layer was washed with brine, dried over Na_2_SO_4_, and concentrated to give a white solid. As the next step, potassium carbonate (16 g, 116 mmol) was added to a solution of the white solid in 420 mL of DMF. After stirring at room temperature for 30 min, MeI (5.0 mL, 80.4 mmol) was added, and stirring continued overnight. The reaction was then quenched by water, and the mixture was extracted with MTBE and washed with brine. The obtained organic layer was dried over Na_2_SO_4_, filtered, and concentrated in vacuo. The residue was thereafter purified by silica gel column chromatography (petroleum ether/EtOAc: 10/1→3/1) to obtain compound **6** (19.6 g, 88% over two steps) as a white solid. ^1^H NMR (600 MHz, DMSO-*d*6) δ 7.53–7.43 (m, 3H), 7.40–7.34 (m, 2H), 3.67 (s, 3H), 3.58 (s, 2H), 3.04 (s, 2H), 2.07 (d, *J* = 13.5 Hz, 2H), 1.78 (q, *J* = 7.4 Hz, 2H), 1.44–1.36 (m, 2H), 1.32 (s, 9H), 0.82 (t, *J* = 7.4 Hz, 3H); ^13^C NMR (150 MHz, DMSO-*d*6) δ 172.9, 153.9, 138.8, 130.5, 129.6, 128.9, 78.8, 61.9, 52.0, 32.5, 28.3, 28.0, 9.1; HRMS (ESI) *m*/*z*: [M + H]^+^ calcd for C_21_H_31_N_2_O_5_ 391.2228; found 391.2214.

#### 3.2.2. Synthesis of Methyl 4-(*N*-Phenylpropionamido)piperidine-4-carboxylate (**4**)

A hydrogen chloride solution (59 mL, 236 mmol, 4 M in 1,4-Dioxane) was added to a solution of compound **6** (18.4 g, 47.1 mmol) in 58 mL of methanol. After stirring at room temperature for 2 h, the reaction was concentrated in vacuo. The reaction was thereafter quenched by saturated aqueous NaHCO_3_, and the mixture was extracted with CH_2_Cl_2_. The obtained organic layer was dried over Na_2_SO_4_, filtered, and concentrated in vacuo to obtain compound **4** (13 g, 95%) as a colorless oil. HRMS (ESI) *m*/*z*: [M + H]+ calcd for C_16_H_23_N_2_O_3_ 291.1703; found 291.1690.

#### 3.2.3. Synthesis of 1-(2-Bromoethyl)-4-(phenylmethoxy)benzene (**5**)

Triphenylphosphine (9.7 g, 37.1 mmol) was added to a solution of compound **8** (5 g, 21.9 mmol) and tetrabromomethane (14.5 g, 43.7 mmol) in 168 mL of acetonitrile at 0 °C. After stirring at 0 °C for 10 min, the suspension was warmed to room temperature, and stirring continued for 2 h. The reaction was thereafter quenched by 6.2 mL of Et_3_N and 6.2 mL of methanol. The mixture was then concentrated in vacuo, and the residue was purified by silica gel column chromatography (petroleum ether/EtOAc: 10:1→2:1) to obtain compound **5** (5.66 g, 89%) as a white solid. ^1^H NMR (600 MHz, DMSO-*d*6): δ 7.46–7.42 (m, 2H), 7.41–7.30 (m, 3H), 7.22–7.16 (m, 2H), 6.97–6.91 (m, 2H), 5.07 (s, 2H), 3.67 (t, *J* = 7.3 Hz, 2H), 3.04 (t, *J* = 7.3 Hz, 2H); ^13^C NMR (150 MHz, DMSO-*d*6): δ 157.2, 137.2, 131.1, 129.8, 128.4, 127.8, 127.7, 114.7, 69.1, 37.6, 34.9; HRMS (ESI) *m*/*z*: [M]+ calcd for C_15_H_15_BrO 290.0301; found 290.0293.

#### 3.2.4. Synthesis of Methyl 1-(4-(Benzyloxy)phenethyl)-4-(*N*-phenylpropionamido)-piperidine-4-carboxylate (**3**)

Cesium carbonate (5.8 g, 17.8 mmol) and compound **5** (3.8 g, 13.1 mmol) were added to a solution of ester **4** (3.45 g, 11.9 mmol) in 35 mL of acetonitrile. The suspension temperature was thereafter increased to 80 °C, and stirring continued overnight. As the next step, the mixture was concentrated in vacuo and extracted with CH_2_Cl_2_/H_2_O. The obtained organic layer was then dried over Na_2_SO_4_, filtered, and concentrated in vacuo. The residue was thereafter purified by silica gel column chromatography (petroleum ether/EtOAc: 10/1→3/1) to obtain compound **3** (5.05 g, 85%) as a white solid. HRMS (ESI) *m*/*z*: [M + H]^+^ calcd for C_31_H_37_N_2_O_4_ 501.2748; found 501.2739.

#### 3.2.5. Synthesis of Methyl 1-[2-(4-Hydroxyphenyl)ethyl]-4-[(1-oxopropyl)phenylamino]-4-piperidinecarboxylate (**2**)

A mixture of compound **3** (1.28 g, 2.56 mmol) and Pd/C (150 mg, 10%) in methanol and acetic acid (10/1, *v*/*v*, 11 mL) was stirred in an atmosphere of H_2_ at room temperature for 12 h. The mixture was thereafter filtrated, concentrated in vacuo, and extracted with CH_2_Cl_2_/saturated aqueous NaHCO_3_. As the next step, the organic layer was dried over Na_2_SO_4_, filtered, and concentrated in vacuo. The residue was then purified by silica gel column chromatography (CH_2_Cl_2_/methanol: 100/1→95/1) to obtain compound **2** (0.88 g, 85%) as a white solid. ^1^H NMR (600 MHz, Chloroform-*d*) δ 7.38–7.34 (m, 3H), 7.26–7.25 (m, 2H), 6.95 (d, *J* = 8.4 Hz, 2H), 6.67 (d, *J* = 8.4 Hz, 2H), 3.79 (s, 3H), 2.88–2.74 (m, 2H), 2.70–2.65 (m, 2H), 2.61–2.44 (m, 4H), 2.38–2.22 (m, 2H), 1.87 (q, *J* = 7.4 Hz, 2H), 1.76–1.66 (m, 2H), 0.95 (t, *J* = 7.4 Hz, 3H); ^13^C NMR (150 MHz, Chloroform-*d*) δ 174.6, 174.1, 155.1, 139.2, 130.6, 129.8, 129.5, 129.0, 115.7, 62.7, 60.6, 52.4, 50.1, 33.1, 32.5, 29.2, 9.4; HRMS (ESI) *m*/*z*: [M + H]+ calcd for C_24_H_31_N_2_O_4_ 411.2278; found 411.2261.

#### 3.2.6. Synthesis of (3*R*,4*S*,5*S*,6*S*)-2-((5-(*tert*-Butyl)-2-methylphenyl)thio)-6-(methoxycarbonyl)-tetrahydro-2*H*-pyran-3,4,5-triyl Triacetate (**12**)

Boron trifluoride diethyl etherate (9.8 mL, 79.7 mmol) was added to a solution of ester **13** (10 g, 26.5 mmol) and 5-tert-butyl-2-methylbenzenethiol (7.33 mL, 39.8 mmol) in 200 mL of CH_2_Cl_2_. After stirring at room temperature for 12 h, the reaction was quenched by Et_3_N, and the mixture was concentrated in vacuo. As the next step, the residue was purified by silica gel column chromatography (petroleum ether/EtOAc: 10:1→3:1) to obtain compound **12** (7.89 g, 60%) as a yellow oil. ^1^H NMR (600 MHz, DMSO-*d*6) δ 7.51–7.14 (m, 3H), 5.81–5.24 (m, 2H), 5.19–5.12 (m, 1H), 5.06–4.59 (m, 2H), 3.63 (d, *J* = 7.2 Hz, 3H), 2.28 (d, *J* = 48.9 Hz, 3H), 2.11–2.02 (m, 6H), 1.97 (d, *J* = 11.1 Hz, 3H), 1.24 (d, *J* = 16.0 Hz, 9H); ^13^C NMR (150 MHz, DMSO-*d*6) δ 169.6, 169.4, 169.2, 169.1, 169.0, 149.4, 149.2, 136.7, 135.3, 131.1, 130.6, 130.2, 129.9, 127.9, 125.5, 124.6, 84.0, 83.6, 74.1, 72.2, 69.6, 69.3, 69.2, 69.0, 68.6,68.4, 52.6, 52.5, 34.2, 34.1, 31.0, 30.9, 30.8, 20.5, 20.4, 20.3, 20.2, 19.6; HRMS (ESI) *m*/*z*: [M + NH4]^+^ Calcd for C_24_H_36_NO_9_S 514.2105; found 514.2105.

#### 3.2.7. Synthesis of Benzyl (2*S*,3*S*,4*S*,5*R*)-3,4,5-*tris*(Benzyloxy)-6-((5-(*tert*-butyl)-2-methylphenyl)thio)tetrahydro-2*H*-pyran-2-carboxylate (**10**)

A sodium hydroxide solution (9.6 mL, 1 M in H2O) was added to a solution of compound **12** (7.89 g, 15.9 mmol) in 4.8 mL of methanol and 4.8 mL of THF. After stirring at room temperature for 5 h, the reaction mixture was neutralized with Amberlite IR120 H+ resin. After filtration of the mixture, the filtrate was concentrated in vacuo. As the next step, NaH (5.08 g, 127 mmol, 60% in oil) and BnBr (21.7 g, 127 mmol) were added to a solution of residue **11** in 150 mL of DMF. This was performed at a temperature of 0 °C. The temperature of the reaction mixture was thereafter allowed to increase to room temperature, and stirring continued overnight. The reaction was then quenched by 1 N HCl, and the mixture was extracted with CH_2_Cl_2_. Thereafter, the organic layer was dried over Na_2_SO_4_. As the next step, the mixture was filtered and concentrated in vacuo to obtain a yellow oil. BnBr (5.4 g, 31.8 mmol) was thereafter added to a solution of this yellow oil in a TBAF solution (70 mL, 1 M in THF) at room temperature. After stirring at room temperature for 12 h, the reaction was quenched by 1 N HCl, and the mixture was extracted with EtOAc and washed with brine. The organic layer was then dried over Na_2_SO_4_, filtered, and concentrated in vacuo. The residue was purified by silica gel column chromatography (petroleum ether/EtOAc: 15/1→10/1) to obtain compound **10** (7.1 g, 62% over two steps) as a yellow oil. HRMS (ESI) *m*/*z*: [M + H]+ calcd for C_45_H_49_O_6_S 717.3244; found 717.3252.

#### 3.2.8. Synthesis of 2,3,4-Tri-*O*-benzyl-d-glucopyranuronic Acid Benzyl Ester (**9**)

NBS (7.06 g, 39.6 mmol) was added to a solution of compound **10** (7.1 g, 9.91 mmol) in acetone and H_2_O (15/1, *v*/*v*, 90 mL). After stirring at room temperature for 5 h, the reaction was quenched by saturated aqueous NaHCO_3_, and the mixture was extracted with CH_2_Cl_2_. Thereafter, the organic layer was dried over Na_2_SO_4_, filtered, and concentrated in vacuo. The residue was then purified by silica gel column chromatography (petroleum ether/EtOAc: 3:1→1:1) to obtain compound **9** (5.4 g, 98%) as a yellow solid. ^1^H NMR (600 MHz, DMSO-*d*6) δ 7.45–7.19 (m, 18H), 7.17–6.95 (m, 2H), 5.33–5.02 (m, 3H), 4.93–4.38 (m, 6H),4.35–3.59 (m, 1H), 3.53–3.24 (m, 4H); ^13^C NMR (150 MHz, DMSO-*d*6) δ 169.3, 168.6, 167.2, 138.7, 138.5, 138.1, 138.0, 137.9, 135.4, 135.3, 128.9, 128.4, 128.3, 128.2, 127.8, 127.7, 127.6, 127.5, 127.4, 97.0, 95.0, 93.4, 90.5, 84.5, 82.9, 82.7, 80.3, 79.9, 79.6, 79.4, 79.2, 75.0, 74.6, 74.5,74.0, 73.9, 73.6, 73.41, 71.4, 69.7, 67.2, 66.5, 53.0; HRMS (ESI) *m*/*z*: [M + Na]+ Calcd for C_34_H_34_O_7_Na 577.2197; found 577.2209.

#### 3.2.9. Synthesis of 1-*O*-Acetyl-2,3,4-tri-*O*-benzyl-d-glucopyranuronic Acid Benzyl Ester (**9-OAc**)

Ac2O (24.4 mg, 0.239 mmol) and DMAP (29.2 mg, 0.239 mmol) were added to a solution of compound **9** (120 mg, 0.217 mmol) in 5 mL of CH_2_Cl_2_. After stirring at room temperature for 6 h, the reaction was quenched by saturated aqueous NaHCO_3_, and the mixture was extracted with CH_2_Cl_2_. Furthermore, the organic layer was dried over Na_2_SO_4_, filtered and then concentrated in vacuo. The residue was thereafter purified by silica gel column chromatography (petroleum ether/EtOAc: 10:1→3:1) to obtain compound **9-OAc** (101 mg, 78%) as a yellow oil. ^1^H NMR (600 MHz, DMSO-*d*6) δ 7.35–7.25 (m, 18H), 7.14–7.07 (m, 2H), 6.40–5.61 (m,1H), 5.26–5.06 (m, 2H), 4.91–4.56 (m, 5H), 4.52–4.17 (m, 2H), 3.95–3.52 (m, 3H), 2.16–2.02 (m, 3H); ^13^C NMR (150 MHz, DMSO-*d*6) δ 169.2, 169.0, 168.2, 168.0, 138.4, 138.3, 138.2, 138.0, 137.7, 135.2, 128.6, 128.5, 128.4, 128.3, 128.2, 128.1, 127.8, 127.7, 127.6, 127.5, 93.0, 89.2, 82.4, 80.3, 80.2, 79.0, 78.5, 77.9, 74.8, 74.6, 74.2, 74.1, 74.0, 73.7, 72.2, 72.0, 66.9, 66.8, 20.7, 20.6; HRMS (ESI) *m*/*z*: [M + Na]+ calcd for C_36_H_36_O_8_Na 619.2302; found 619.2316.

#### 3.2.10. Synthesis of Methyl 4-(*N*-Phenylpropionamido)-1-(4-(((3*R*,4*S*,5*S*,6*S*)-3,4,5-*tris*(benzyloxy)-6-((benzyloxy)carbonyl)tetrahydro-2*H*-pyran-2-yl)oxy)phenethyl)piperidine-4-carboxylate (**15**)

Trichloroacetonitrile (2.66 g, 18.4 mmol) was added to a solution of compound **9** (2.04 g, 3.68 mmol) in 10 mL of anhydrous CH_2_Cl_2_. This was performed at a temperature of 0 °C. After stirring at 0 °C for 15 min, DBU (151 mg, 1.0 mmol) was added to the mixture, and stirring continued overnight. The reaction was thereafter concentrated in vacuo. The residue was then purified by silica gel column chromatography (petroleum ether/EtOAc: 10:1→3:1) to obtain compound **14** (2.05 g, 80%) as a colorless oil. As the next step, BF_3_.Et_2_O (414 mg, 2.93 mmol) was added to a solution of the colorless oil (2.05 g, 2.93 mmol), compound **2** (401 mg, 0.97 mmol), and freshly activated 4 Å MS (3 g) in 15 mL of anhydrous toluene. This was performed at a temperature of 0 °C and in a nitrogen atmosphere. The reaction mixture was thereafter allowed to reach room temperature. After stirring at room temperature for 24 h, the mixture was quenched by Et_3_N, filtered, and concentrated in vacuo. As the next step, the residue was purified by silica gel column chromatography (CH2Cl2/methanol: 100/1→10/1) to obtain compound **15** (750 mg, 81%) as a colorless syrup. HRMS (ESI) *m*/*z*: [M + H]+ calcd for C_58_H_63_N_2_O_10_ 947.4477; found 947.4489.

#### 3.2.11. Synthesis of (2*S*,3*S*,4*S*,5*R*)-3,4,5-Trihydroxy-6-(4-(2-(4-(methoxycarbonyl)-4-(*N*-phenylpropionamido)-piperidin-1-yl)ethyl)phenoxy)tetrahydro-2*H*-pyran-2-carboxylic Acid (**1**)

A mixture of compound **15** (510 mg, 0.538 mmol) and Pd/C (200 mg, 10%) in methanol and acetic acid (20/1, *v*/*v*, 10.5 mL) was stirred in an atmosphere of H_2_ at room temperature for 36 h. The following filtration, concentration in vacuo, and elution resulted in compound **1** (126 mg, 40%) as a white solid. ^1^H NMR (600 MHz, D2O) δ 7.59–7.34 (m, 5H), 7.19–6.95 (m, 4H), 5.65–4.97 (m, 1H), 4.03–3.65 (m, 5H), 3.61–2.70 (m, 10H), 2.58–2.31 (m, 2H), 2.07–1.70 (m, 4H), 0.87 (t, *J* = 7.4 Hz, 3H); ^13^C NMR (150 MHz, D2O) δ 177.9, 176.2, 175.4, 174.3, 155.4, 155.1, 137.2, 130.8, 130.7, 130.2, 130.1, 130.0, 129.9, 129.8, 117.7, 116.5, 99.5, 97.2, 76.1, 75.3, 72.8, 72.7, 71.9, 71.7, 70.9, 60.8, 57.1, 53.2, 49.2, 29.9, 29.0, 28.6, 8.4; HRMS (ESI) *m*/*z*: [M + H]+ calcd for C_30_H_39_N_2_O_10_ 587.2599; found 587.2590.

## 4. Conclusions

In the present study, a method for the synthesis of the first-phase and second-phase metabolites of carfentanil has been provided. By using this method, the first-phase metabolite **2** (with a total yield of 30%) and the biphasic phase metabolite **1** (with a total yield of 9.7%) of carfentanil were successfully produced in 6 and 8 steps, respectively. By combining 1H NMR and 13C NMR analyses, it was confirmed that the biphasic metabolite was a mixture of 1α and 1β anomers. Due to the lack of reports on the specific structures of these two metabolites, the samples produced here can be used for comparison purposes. They will also provide sufficient references for the synthesis of similar compounds in the future. In future studies by the present research group, the metabolism of carfentanil in liver microsomes will be simulated, and the reference substances will be used for the metabolism of carfentanil. HPLC, HRMS, and LC-MS techniques will then be used in the analysis and detection of the metabolites of carfentanil, with the purpose of further validating the metabolic pathway of carfentanil.

## Figures and Tables

**Table 1:** Optimization of reaction conditions.

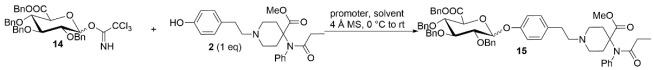				
Entry	Compound 14 (Eq.)	Promoter (Eq.)	Solvent	Yield ^a^
1	1	TMSOTf (0.3) ^b^	CH_2_Cl_2_	8%
2	1	TBSOTf (0.3) ^c^	CH_2_Cl_2_	13%
3	1	BF_3_.Et_2_O (0.3)	CH_2_Cl_2_	22%
4	1	BF_3_.Et_2_O (0.6)	CH_2_Cl_2_	32%
5	1	BF_3_.Et_2_O (1)	CH_2_Cl_2_	51%
6	1	BF_3_.Et_2_O (1)	toluene	58%
7	2	BF_3_.Et_2_O (2)	toluene	72%
8	3	BF_3_.Et_2_O (3)	toluene	81%

^a^ Isolated yield. ^b^ TMSOTf: trimethylsilyl trifluromethanesulfonate. ^c^ TBSOTf: tert-butyldimethylsilyl trifluoromethane.

## Data Availability

Data are contained within the article and Supplementary Materials.

## Supplementary Materials

The following supporting information can be downloaded at: https://www.mdpi.com/article/10.3390/molecules28227625/s1. NMR and HRMS spectra for eleven products (compound **12**, compound **11**, compound **10**, compound **9**, compound **9-OAc**, compound **6**, compound **5**, compound **4**, compound **3**, compound **2**, and compound **1**).
