# Supplementary material for: The Synthesis of Biphasic Metabolites of Carfentanil
Source: Molecules. 2023 Nov 16;28(22):7625. doi: 10.3390/molecules28227625 (PMC10674982; doi:10.3390/molecules28227625)
Supplement: Supplementary file 1 [file molecules-28-07625-s001.zip › molecules-2652757-supplementary.pdf]

# Supporting Information

# The Synthesis of Biphase Metabolites of Carfentanil

## contents

Figure S1. Compound 6 $^{1}H$ NMR ........ 2
Figure S2. Compound 6 $^{13}C$ NMR ........ 2
Figure S3. Compound 6 HRMS ........ 3
Figure S4. Compound 4 HRMS ........ 3
Figure S5. Compound 5 $^{1}H$ NMR ........ 4
Figure S6. Compound 5 $^{13}C$ NMR ........ 4
Figure S7. Compound 5 HRMS ........ 5
Figure S8. Compound 3 HRMS ........ 5
Figure S9. Compound 2 $^{1}H$ NMR ........ 6
Figure S10. Compound 2 $^{13}C$ NMR ........ 6
Figure S11. Compound 2 HRMS ........ 7
Figure S12. Compound 12 $^{1}H$ NMR ........ 7
Figure S13. Compound 12 $^{13}C$ NMR ........ 8
Figure S14. Compound 12 HRMS ........ 8
Figure S15. Compound 10 HRMS ........ 9
Figure S16. Compound 9 $^{1}H$ NMR ........ 9
Figure S17. Compound 9 $^{13}C$ NMR ........ 10
Figure S18. Compound 9 HRMS ........ 10
Figure S19. Compound 9-OAc $^{1}H$ NMR ........ 11
Figure S20. Compound 9-OAc $^{13}C$ NMR ........ 11
Figure S21. Compound 9-OAc HMQC ........ 12
Figure S22. Compound 9-OAc HRMS ........ 12
Figure S23. Compound 15 HRMS ........ 13
Figure S24. Compound 1 $^{1}H$ NMR ........ 13
Figure S25. Compound 1 $^{13}C$ NMR ........ 14
Figure S26. Compound 1 HRMS ........ 14

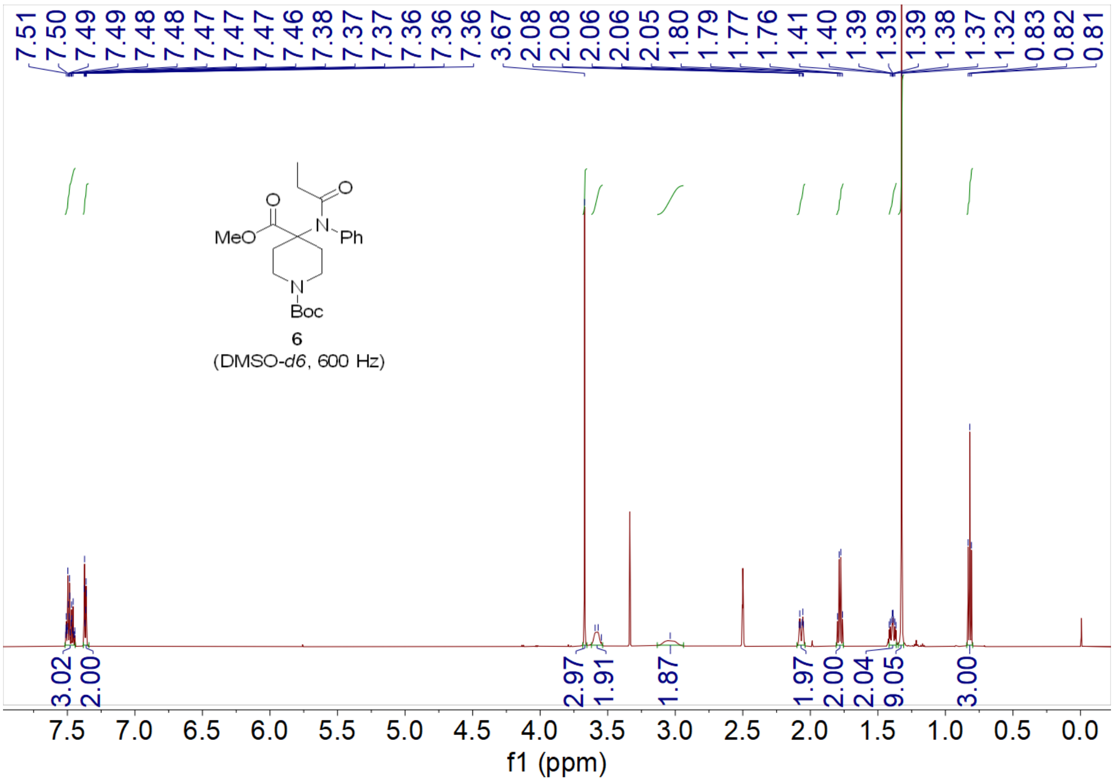


**Figure S1. Compound 6 $^{1}$H NMR**

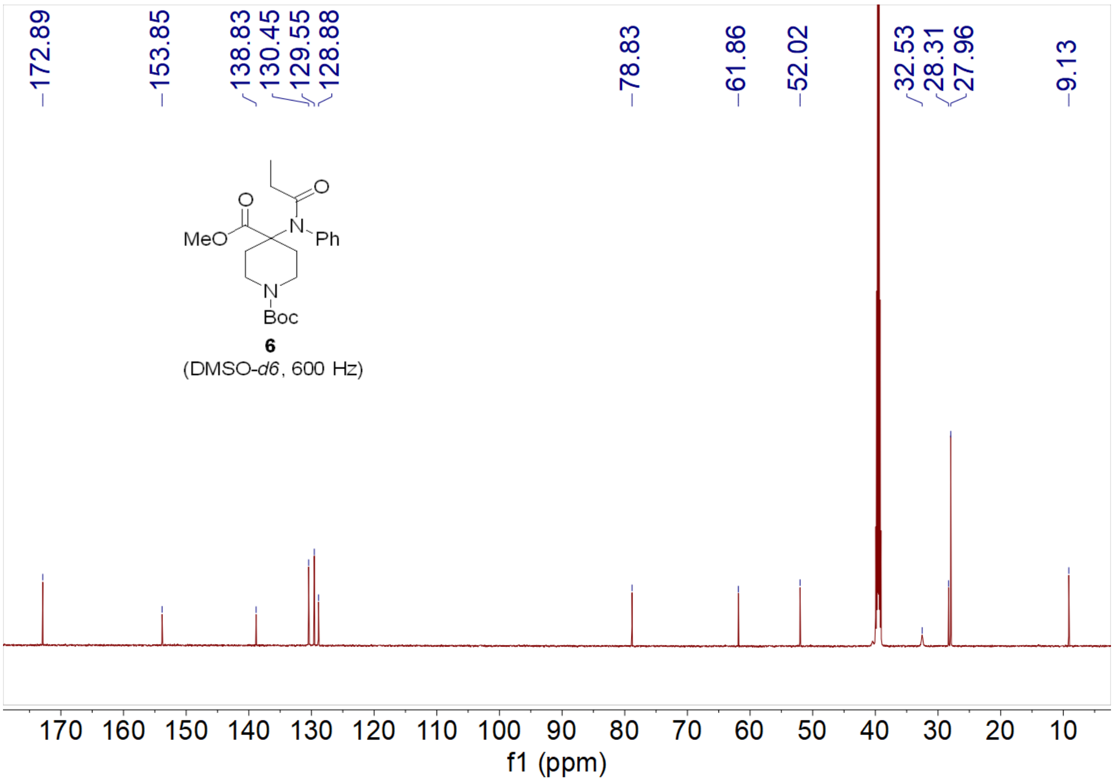


**Figure S2. Compound 6 $^{13}$C NMR**

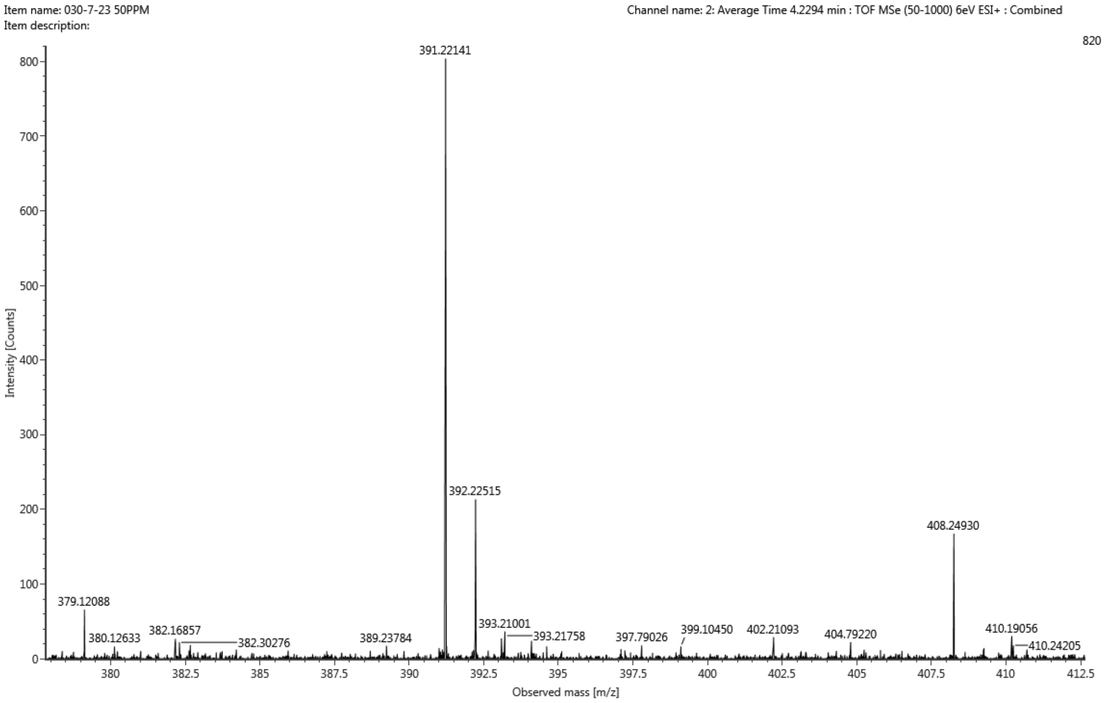


**Figure S3. Compound 6 HRMS**

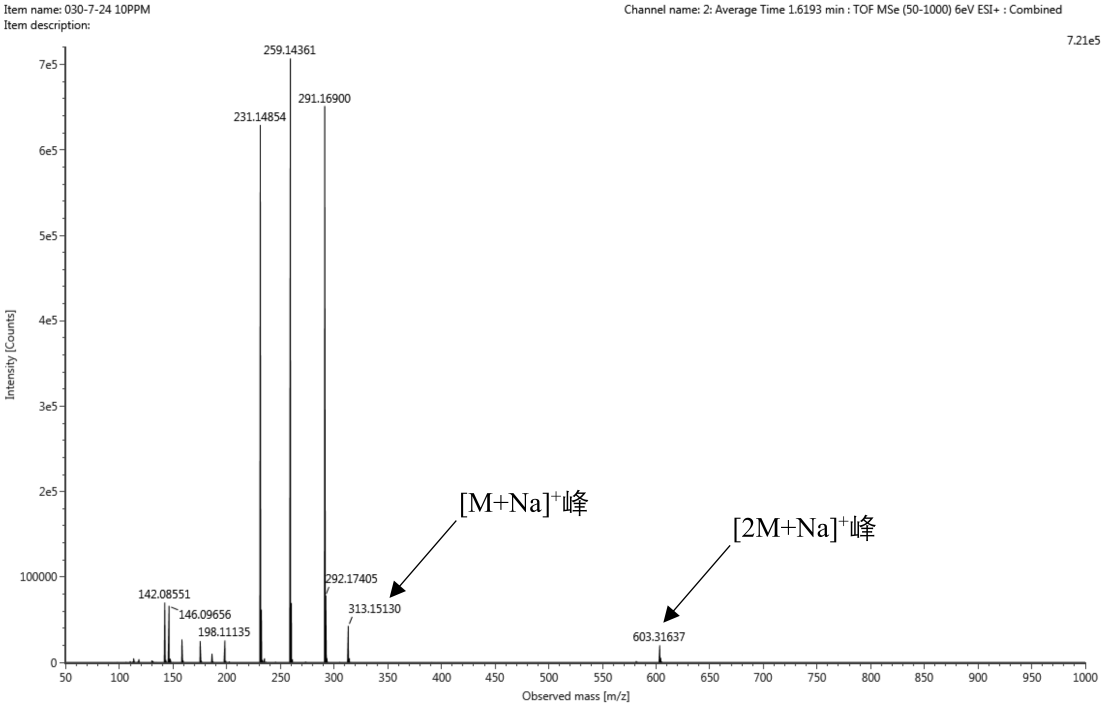


**Figure S4. Compound 4 HRMS**

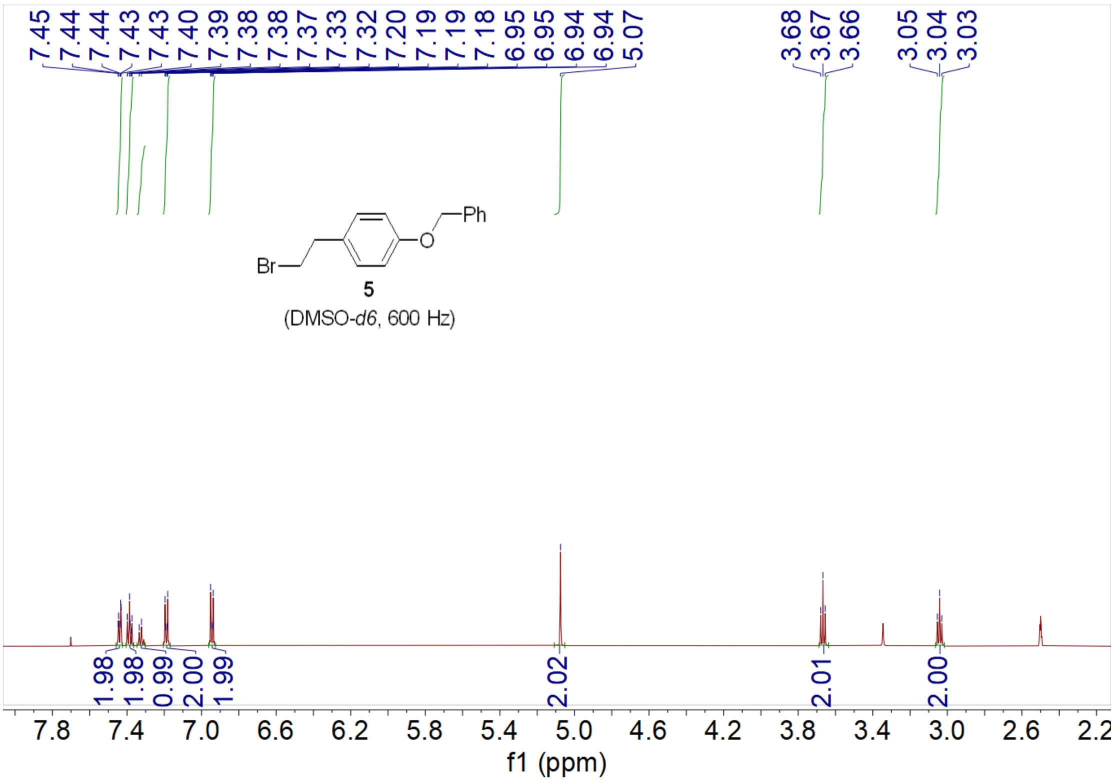


**Figure S5. Compound 5 $^{1}$H NMR**

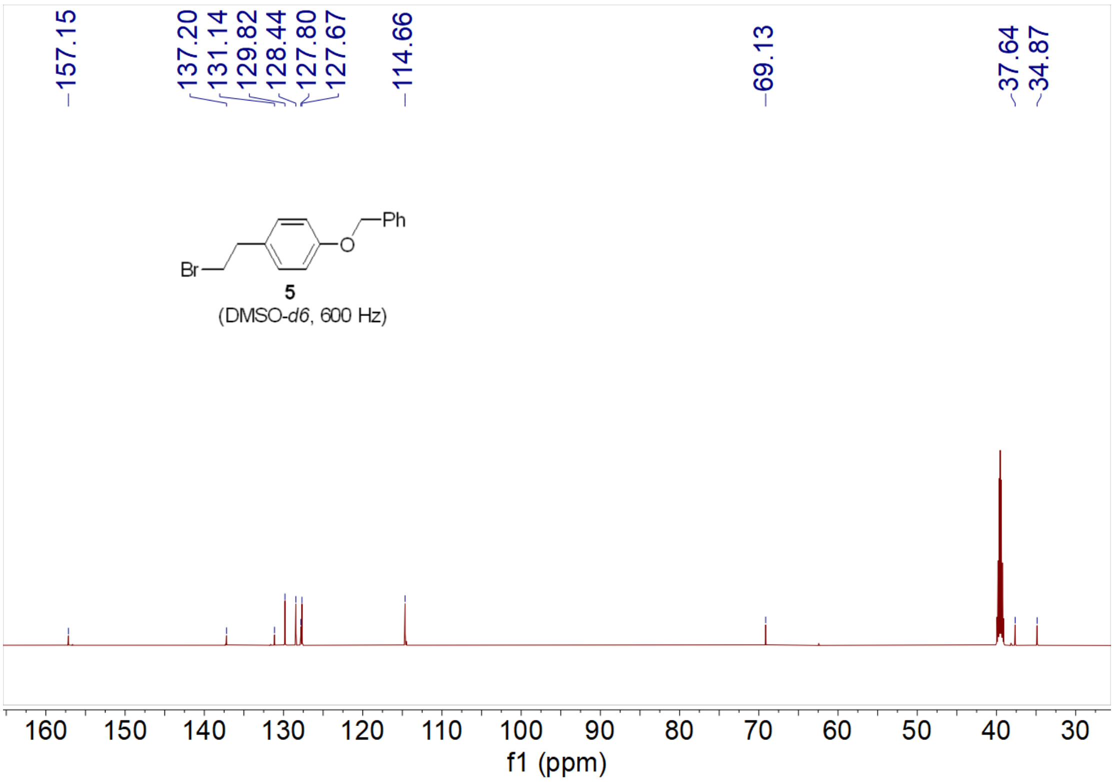


**Figure S6. Compound 5 $^{13}$C NMR**

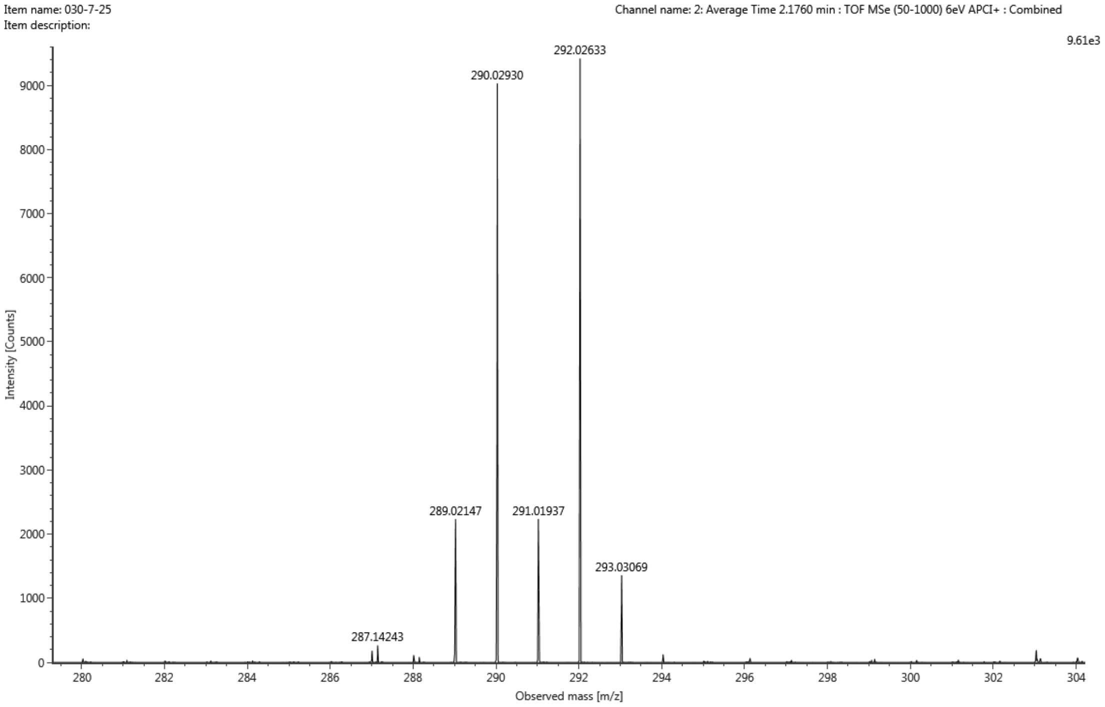


**Figure S7. Compound 5 HRMS**

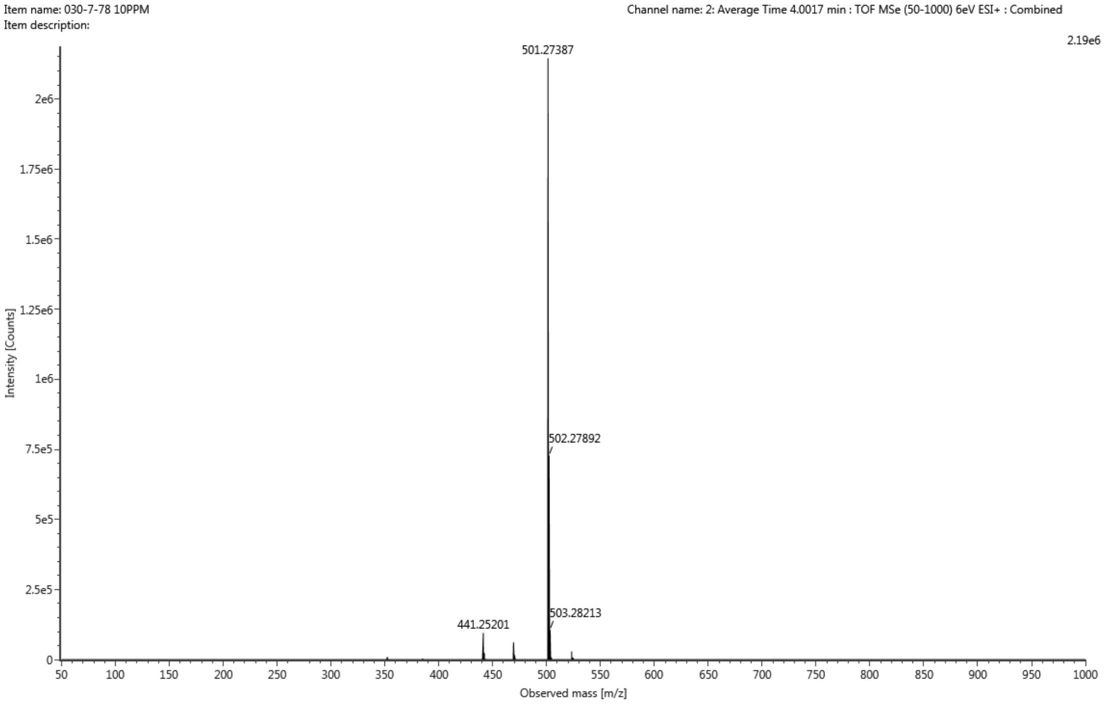


**Figure S8. Compound 3 HRMS**

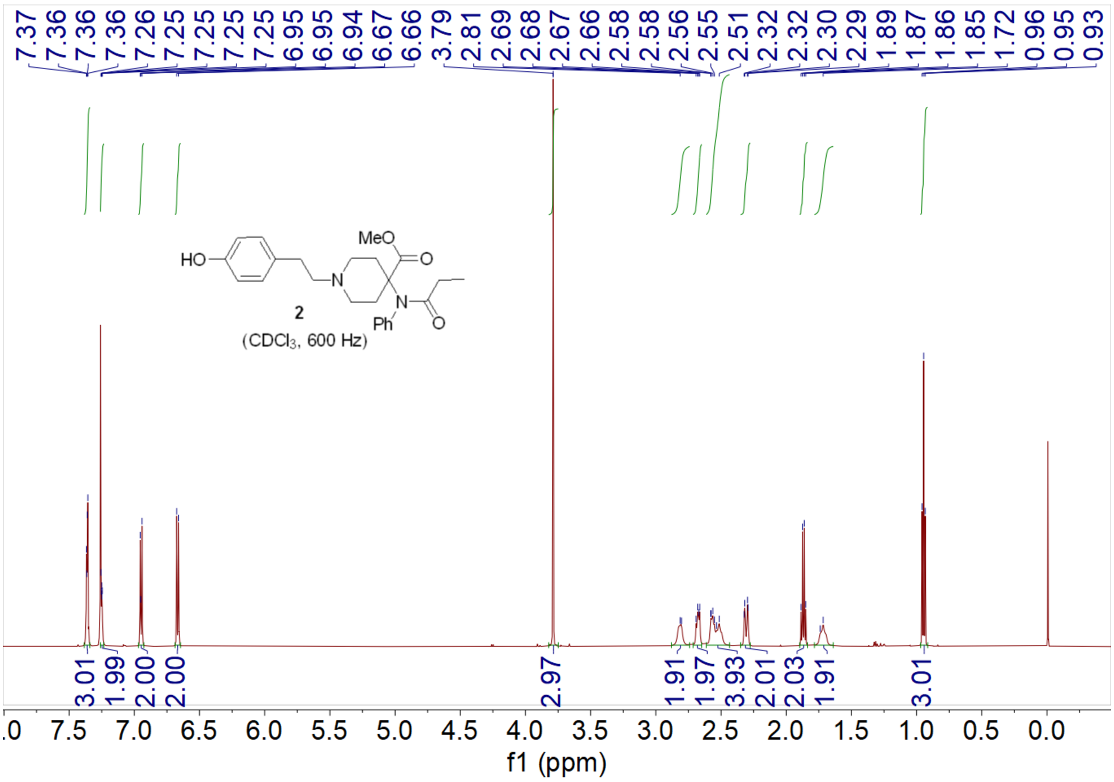


**Figure S9. Compound 2 $^{1}$H NMR**

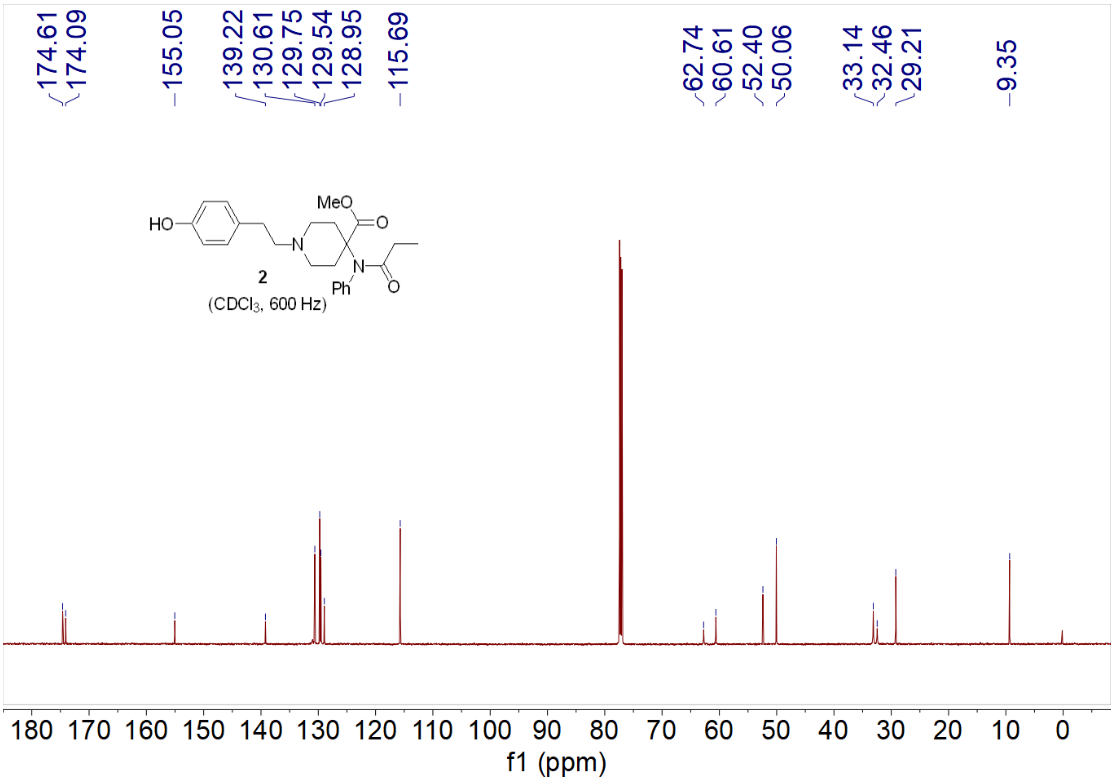


**Figure S10. Compound 2 $^{13}$C NMR**

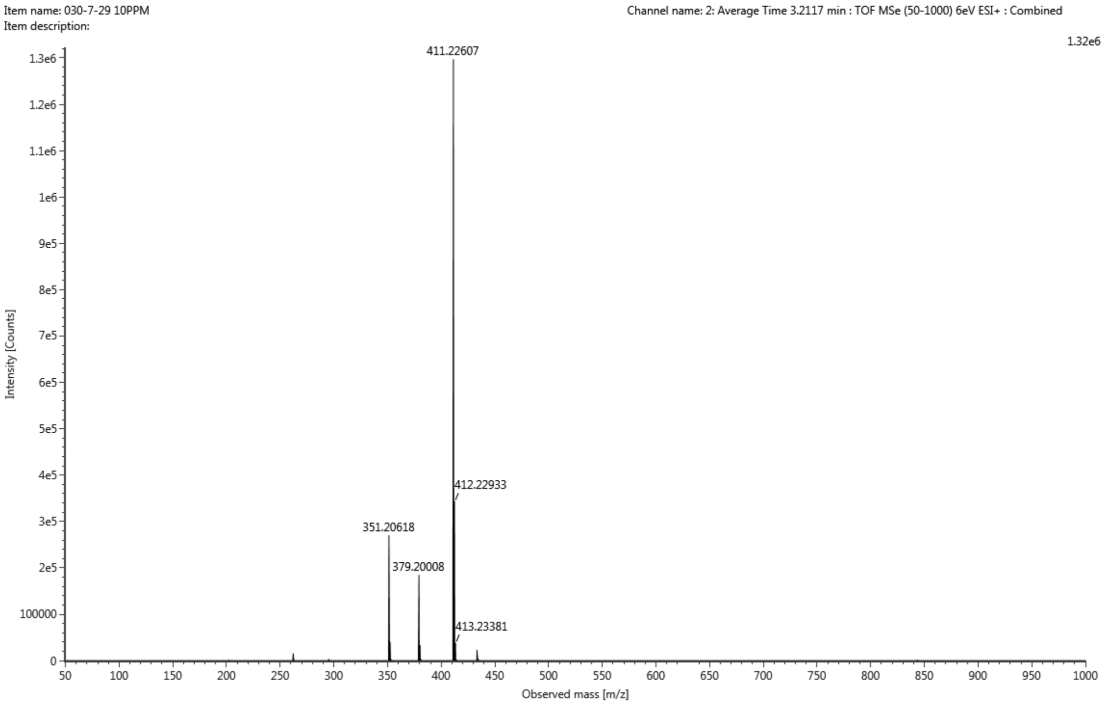


**Figure S11. Compound 2 HRMS**

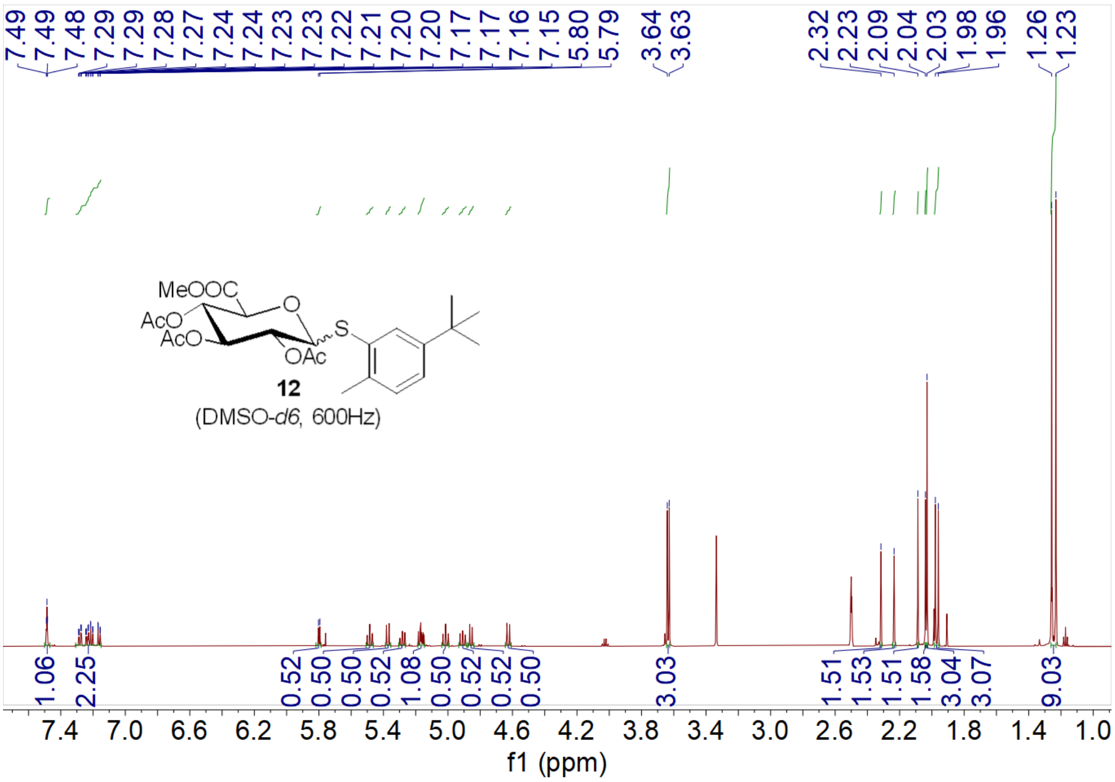


**Figure S12. Compound 12 $^1$H NMR**

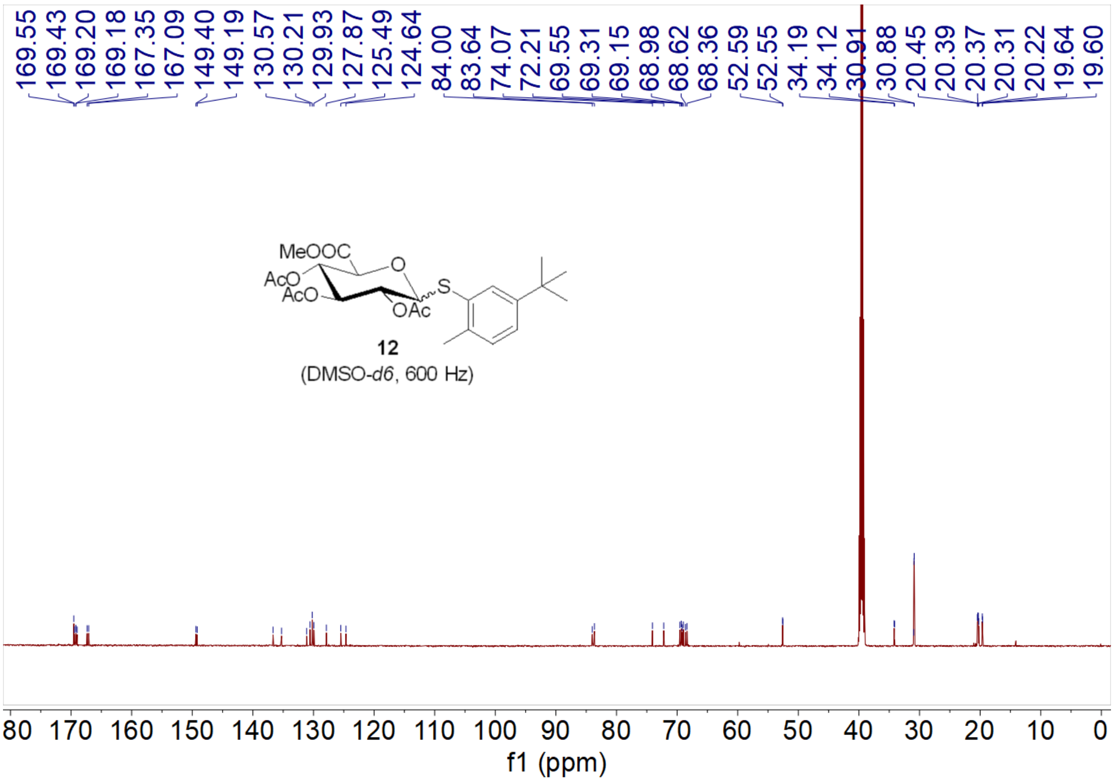


**Figure S13. Compound 12 $^{13}$C NMR**

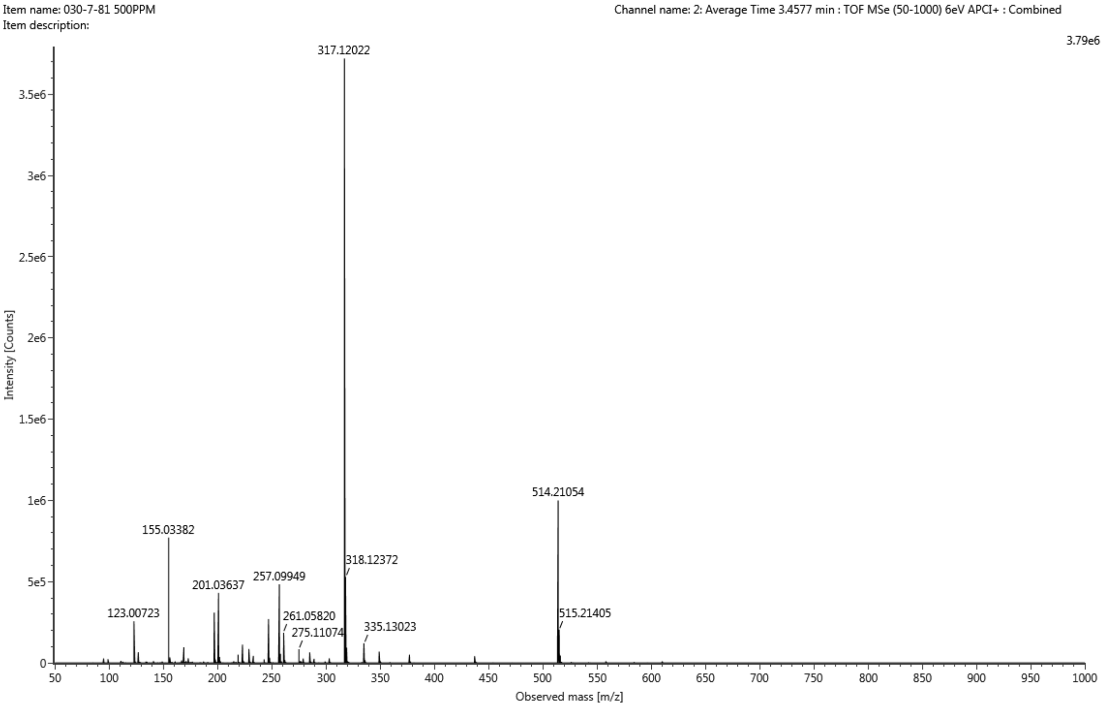


**Figure S14. Compound 12 HRMS**

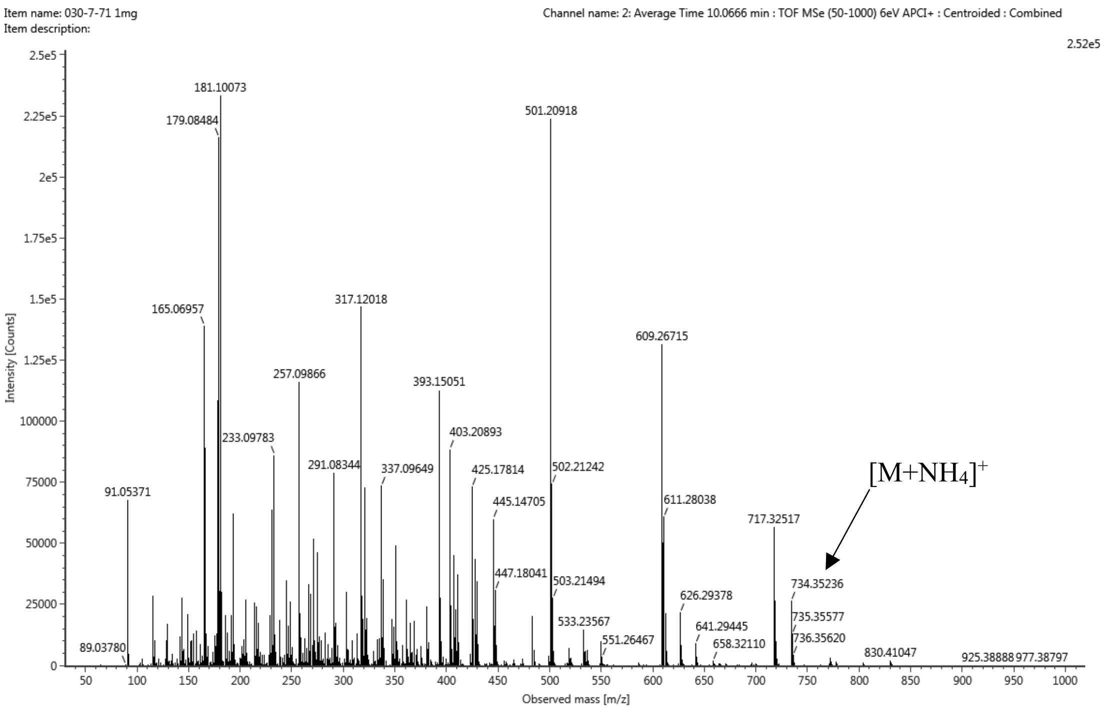


**Figure S15. Compound 10 HRMS**

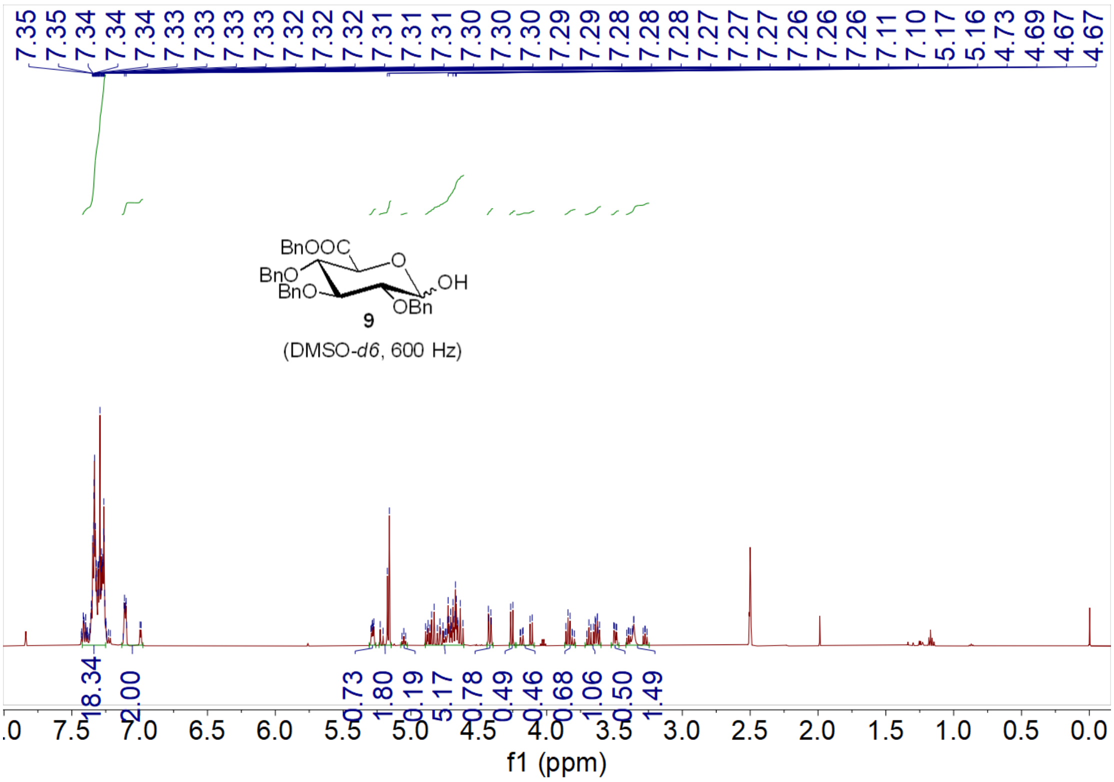


**Figure S16. Compound 9 $^{1}$H NMR**

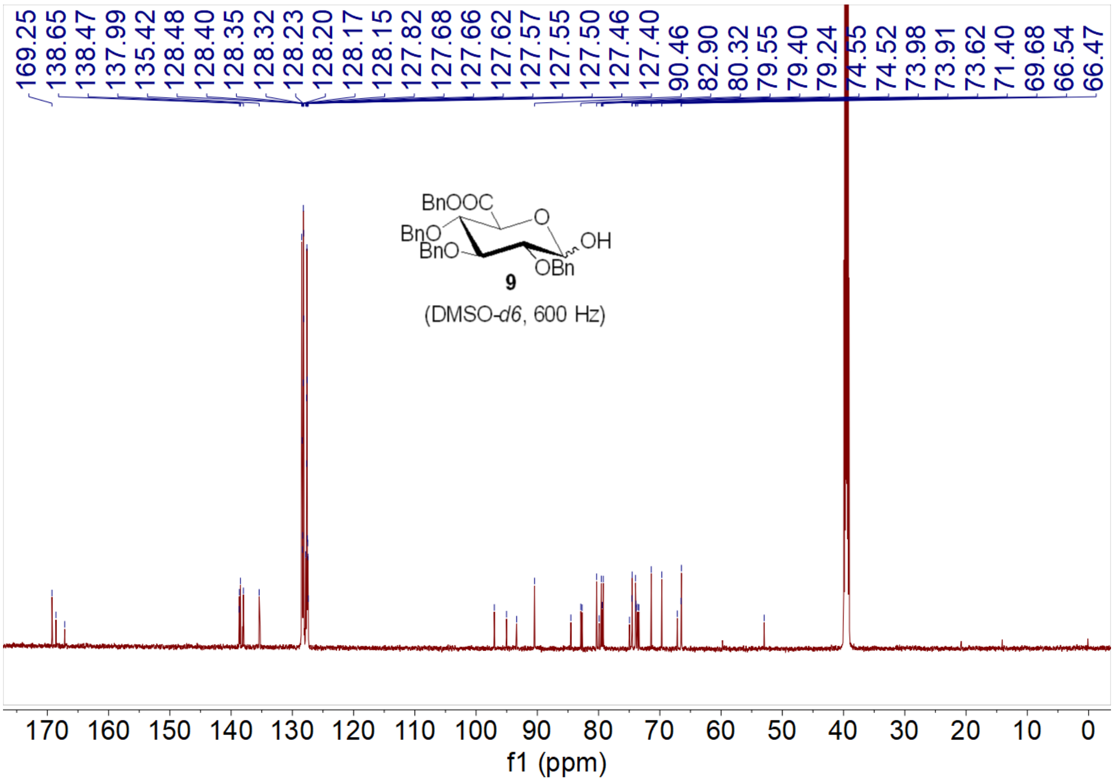


**Figure S17. Compound 9 $^{13}$C NMR**

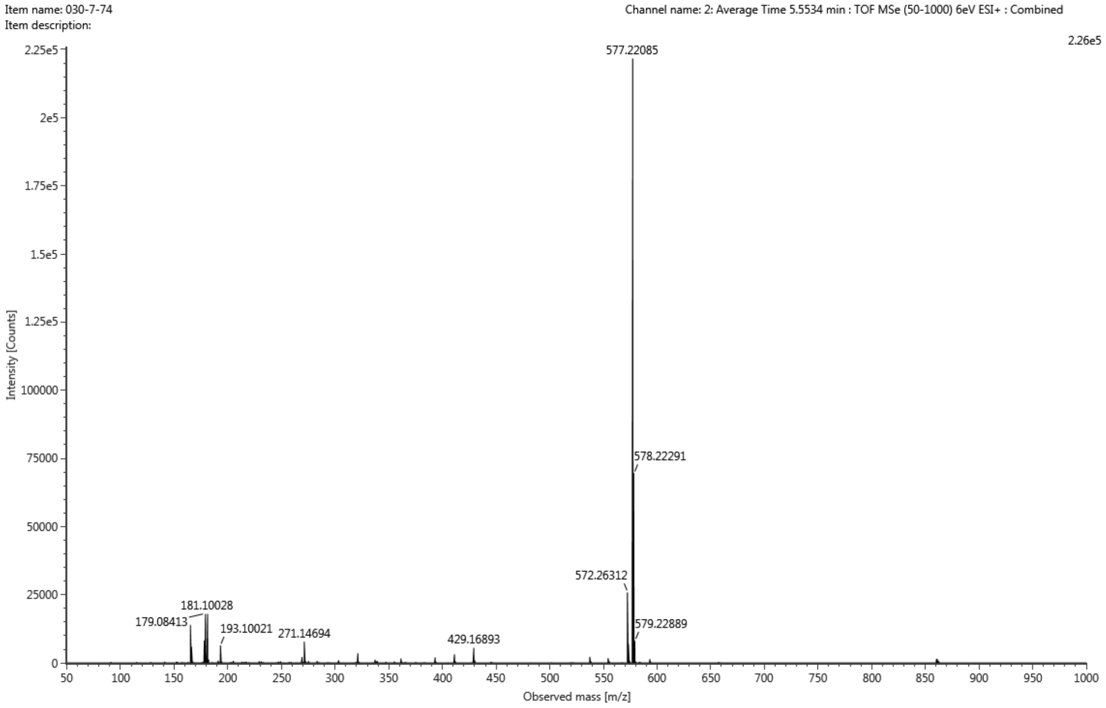


**Figure S18. Compound 9 HRMS**

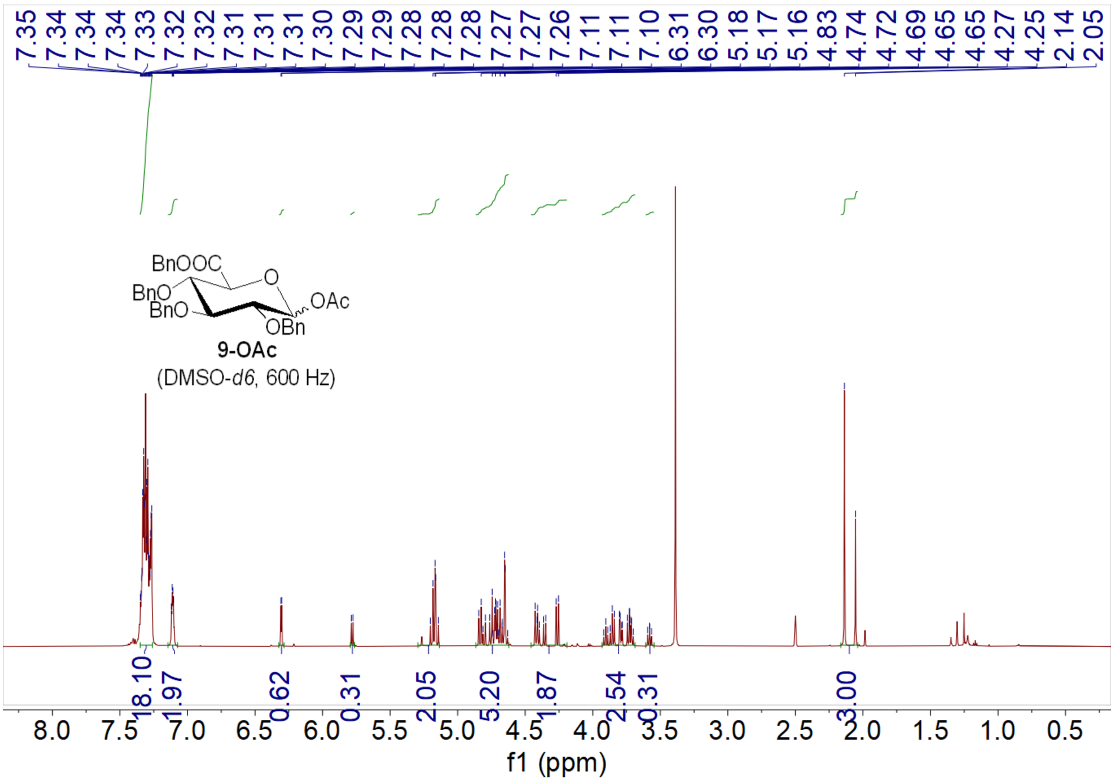


**Figure S19. Compound 9-OAc ¹H NMR**

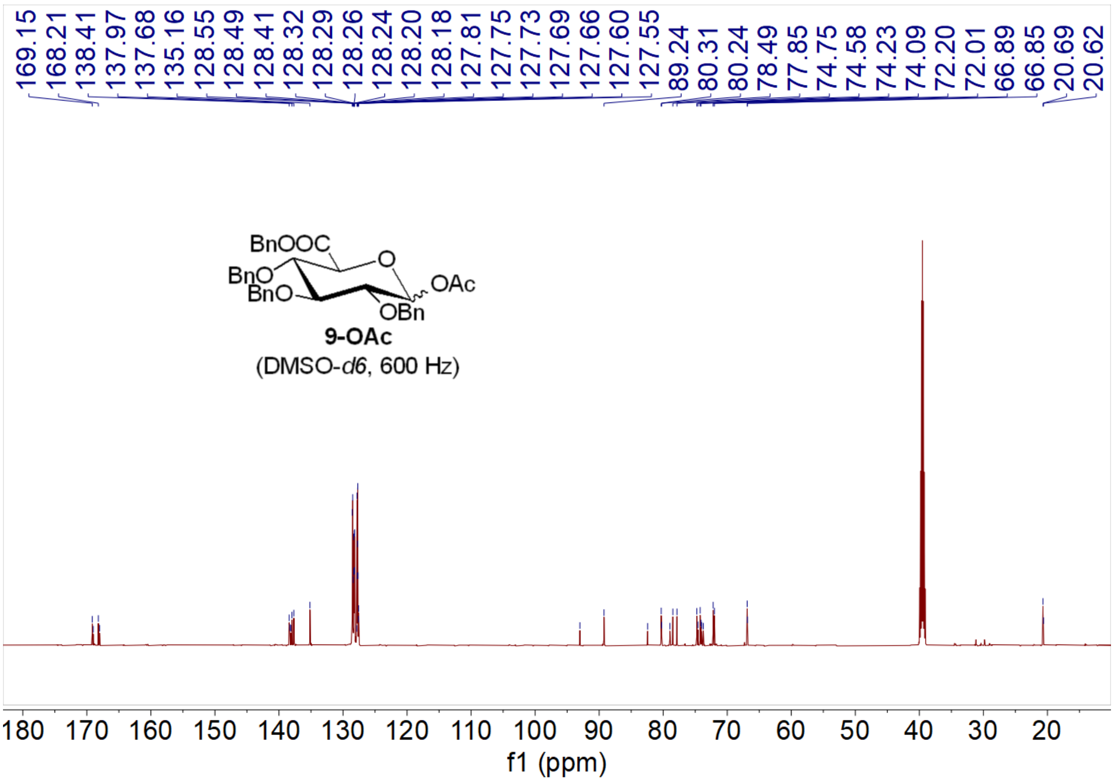


**Figure S20. Compound 9-OAc ¹³C NMR**

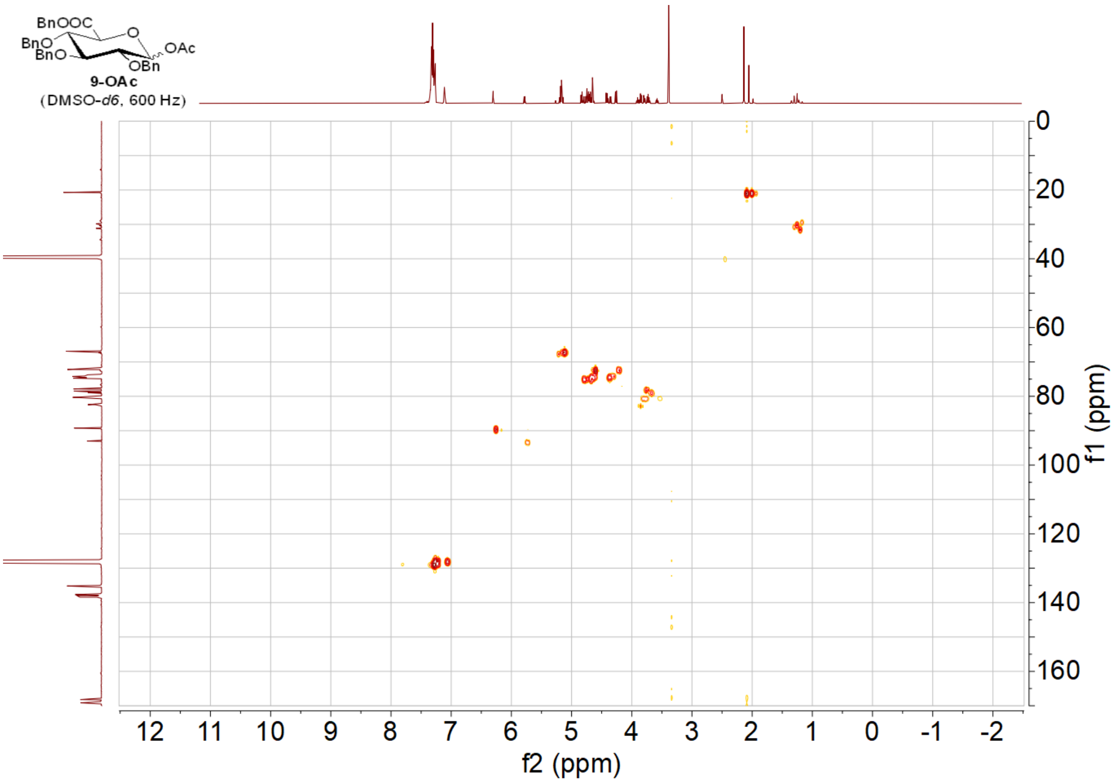


**Figure S21. Compound 9-OAc HMQC**

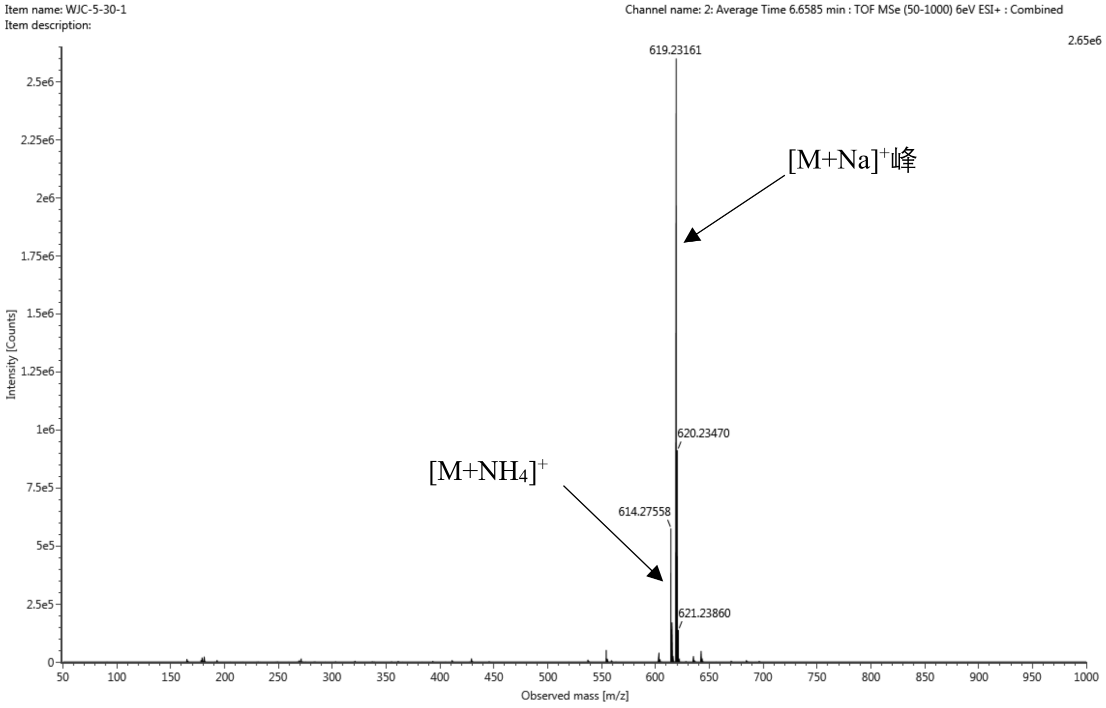


**Figure S22. Compound 9-OAc HRMS**

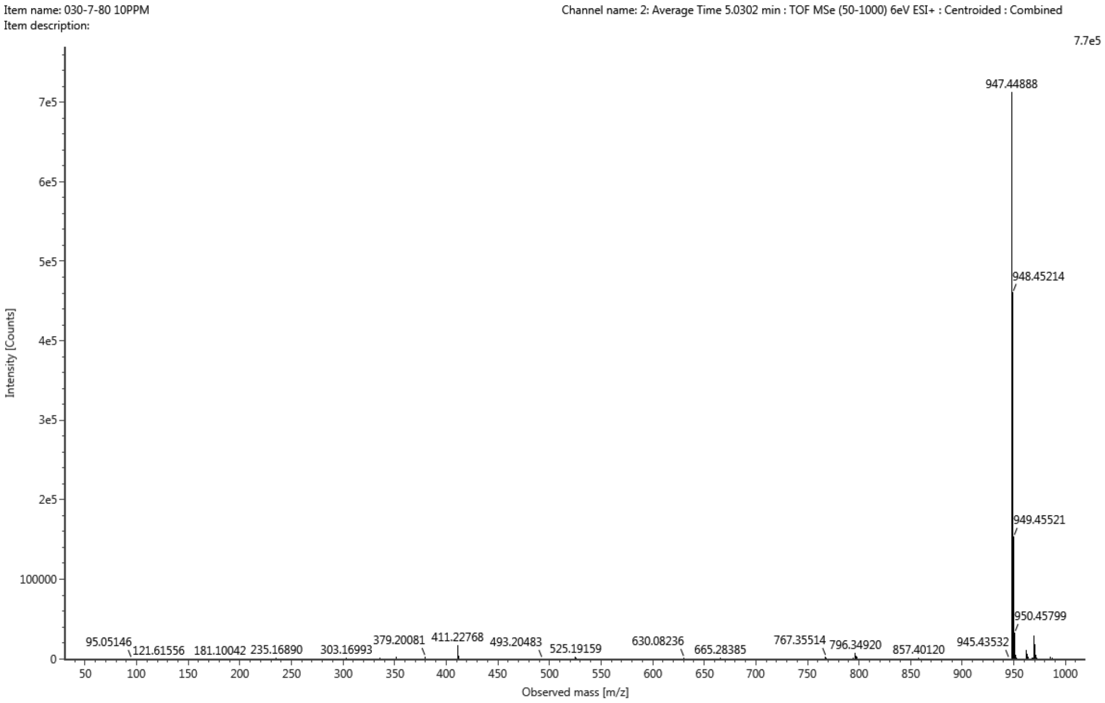


**Figure S23. Compound 15 HRMS**

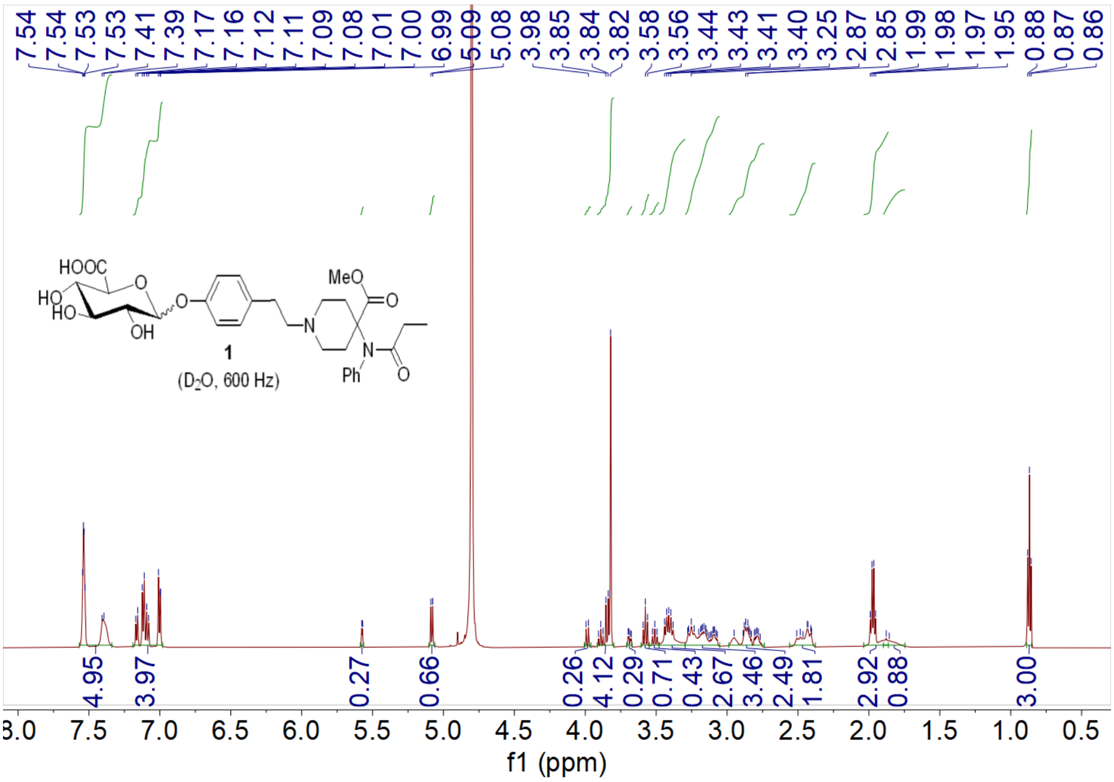


**Figure S24. Compound 1 $^1$H NMR**

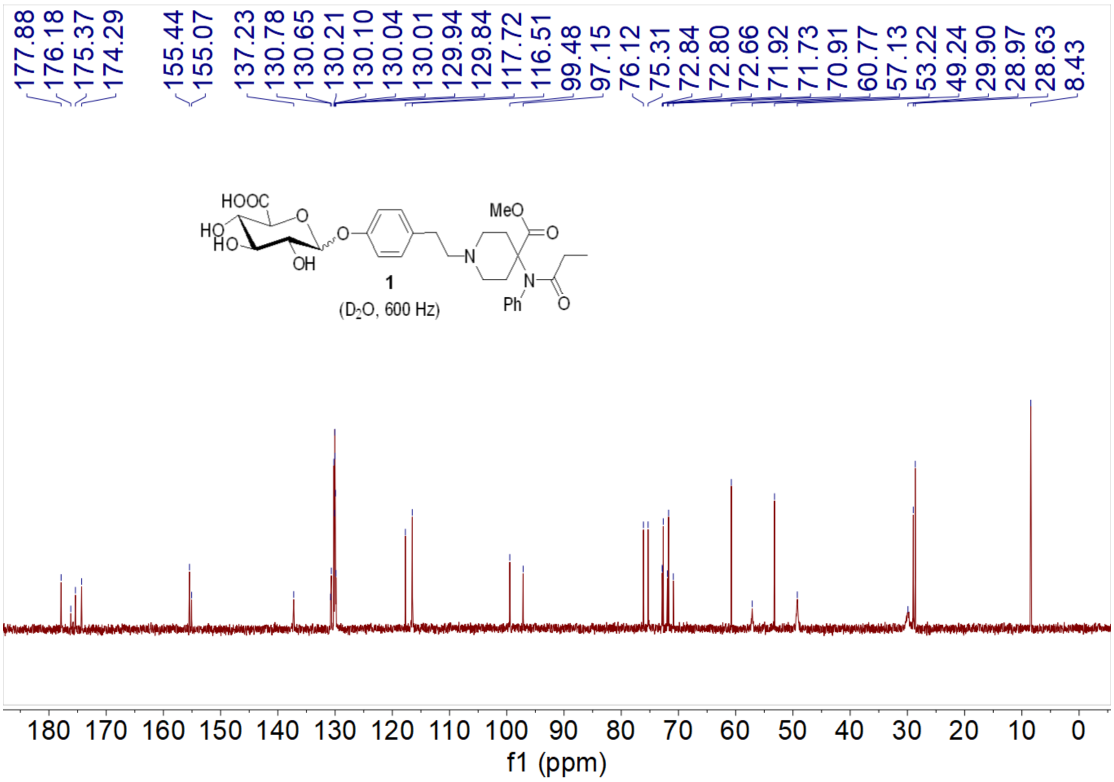


**Figure S25. Compound 1 $^{13}$C NMR**

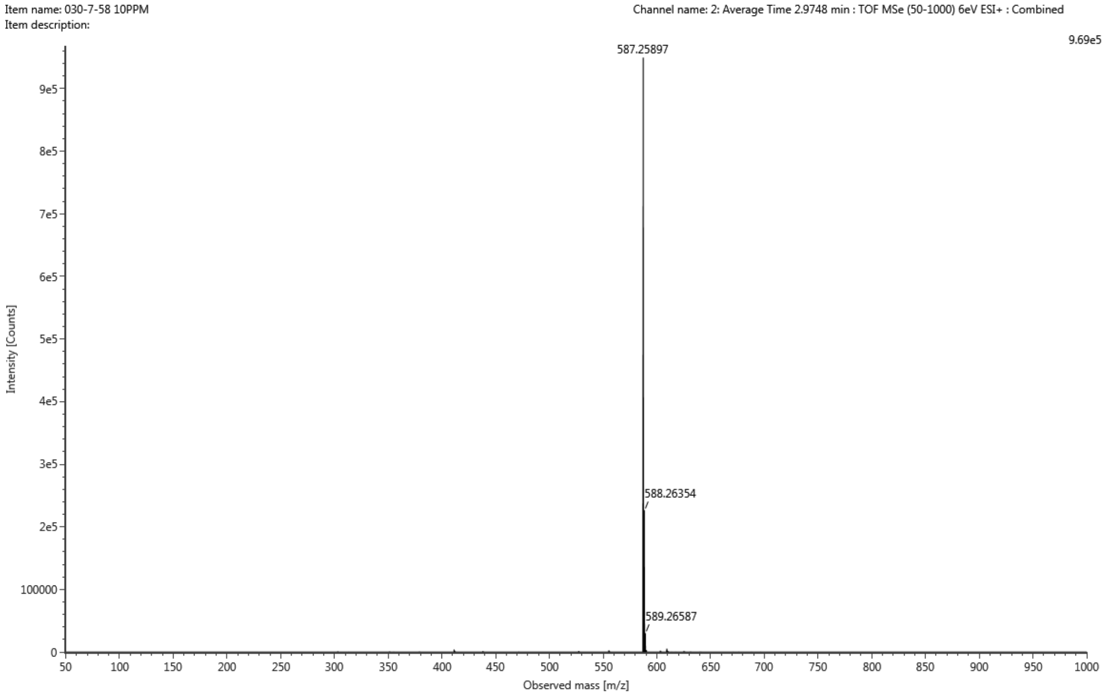


**Figure S26. Compound 1 HRMS**
